# Lipidome of mammographic breast density in premenopausal women

**DOI:** 10.1186/s13058-023-01725-1

**Published:** 2023-10-09

**Authors:** Kayla R. Getz, Myung Sik Jeon, Chongliang Luo, Jingqin Luo, Adetunji T. Toriola

**Affiliations:** 1 Division of Public Health Sciences, Department of Surgery, School of Medicine, Washington University, 660 South Euclid Avenue, Box 8100, St. Louis, MO 63110 USA; 2 Siteman Cancer Center Biostatistics Shared Resource, Division of Public Health Sciences, Department of Surgery, Washington University School of Medicine, St. Louis, MO USA; 3 Siteman Cancer Center, Washington University School of Medicine, St. Louis, MO USA

**Keywords:** Lipidomics, Mammographic breast density, Premenopausal

## Abstract

**Background:**

High mammographic breast density (MBD) is a strong risk factor for breast cancer development, but the biological mechanisms underlying MBD are unclear. Lipids play important roles in cell differentiation, and perturbations in lipid metabolism are implicated in cancer development. Nevertheless, no study has applied untargeted lipidomics to profile the lipidome of MBD. Through this study, our goal is to characterize the lipidome of MBD in premenopausal women.

**Methods:**

Premenopausal women were recruited during their annual screening mammogram at the Washington University School of Medicine in St. Louis, MO. Untargeted lipidomic profiling for 982 lipid species was performed at Metabolon (Durham, NC®), and volumetric measures of MBD (volumetric percent density (VPD), dense volume (DV), and non-dense volume (NDV)) was assessed using Volpara 1.5 (Volpara Health®). We performed multivariable linear regression models to investigate the associations of lipid species with MBD and calculated the covariate-adjusted least square mean of MBD by quartiles of lipid species. MBD measures were log_10_ transformed, and lipid species were standardized. Linear coefficients of MBD were back-transformed and considered significant if the Bonferroni corrected p-value was < 0.05.

**Results:**

Of the 705 premenopausal women, 72% were non-Hispanic white, and 23% were non-Hispanic black. Mean age, and BMI were 46 years and 30 kg/m^2^, respectively. Fifty-six lipid species were significantly associated with VPD (52 inversely and 4 positively). The lipid species with positive associations were phosphatidylcholine (PC)(18:1/18:1), lysophosphatidylcholine (LPC)(18:1), lactosylceramide (LCER)(14:0), and phosphatidylinositol (PI)(18:1/18:1). VPD increased across quartiles of PI(18:1/18:1): (Q1 = 7.5%, Q2 = 7.7%, Q3 = 8.4%, Q4 = 9.4%, Bonferroni p-trend = 0.02). The lipid species that were inversely associated with VPD were mostly from the triacylglycerol (*N* = 43) and diacylglycerol (*N* = 7) sub-pathways. Lipid species explained some of the variation in VPD. The inclusion of lipid species increased the adjusted R^2^ from 0.45, for a model that includes known determinants of VPD, to 0.59.

**Conclusions:**

We report novel lipid species that are associated with MBD in premenopausal women. Studies are needed to validate our results and the translational potential.

**Supplementary Information:**

The online version contains supplementary material available at 10.1186/s13058-023-01725-1.

## Background

High mammographic breast density (MBD) is a strong risk factor for breast cancer development [1]. Women with extremely dense breasts have a 4–sixfold increased risk of developing breast cancer compared to women with scattered areas of fibroglanduar density [1, 2]. It is estimated that ~ 29% of breast cancers in premenopausal women could be potentially prevented if women with dense or heterogeneously dense breasts reduced their density to scattered areas of fibroglandular density [3]. Further, MBD is an intermediate phenotype [4] for breast cancer development; hence, understanding the mechanisms underlying high MBD may open up new avenues for breast cancer prevention.

Adiposity is strongly associated with MBD measures. We and others have demonstrated that childhood, early adulthood, and adulthood adiposity, as well as changes in adiposity over the life course, are associated with MBD in premenopausal women [5–7]. Adiposity explains the greatest percentage of variability in MBD, approximately 22% in non-Hispanic black women and 26% in non-Hispanic white women [8]. Studies on the associations of adiposity biomarkers with MBD yielded inconsistent results [9–12]. Thus, there is an urgent need to investigate the associations of novel biomarkers of adiposity with MBD.

The lipidome is the complete lipid profile within a tissue [13]. Lipidomics, the comprehensive analysis of lipid molecules, is essential to understanding lipid biology. Recent technological advances have allowed the quantification of lipid species unbiasedly with increasing accuracy. Lipids play essential roles in cell structure, generation of cell membranes, signaling, and fuel/storage [14]. Lipids also function as second messengers and as hormones and are responsible for various cell functions. Changes in lipid metabolism have been shown to impact cell growth, proliferation, differentiation, and motility [15], and perturbations in lipid metabolism are implicated in metabolic diseases [16, 17]. Applying lipidomics to MBD should, therefore, provide meaningful insights into the biologic mechanisms underlying MBD, but to the best of our knowledge, no study has characterized the lipidome of MBD. Our study aimed to characterize, for the first time, the comprehensive lipidome of MBD in premenopausal women.

## Methods

### Study population

This study population is comprised of 705 premenopausal women who were recruited during annual screening mammogram at the Joanne Knight Breast Health Center at the Siteman Cancer Center at Washington University School of Medicine (WU) in St. Louis, MO. Women were eligible to participate in the study if they were premenopausal, not pregnant, and were able to comply with study procedures. Women with a history of cancer, breast augmentation (implants or reduction), and currently use or have used selective estrogen receptor modulators in the past six months were excluded [6]. Women were considered premenopausal if they had a regular menstrual cycle in the past 12 months and did not have a history of hormone replacement therapy or bilateral oophorectomy [6]. All participants provided written informed consent, and the study was performed in accordance with the Declaration of Helsinki. We received approval for this study from the WU Institutional Review Board.

Participants completed a questionnaire on behavioral, reproductive, demographic, and clinical characteristics. They also had their height, weight, and body fat percentage measured on the day of their screening mammogram visit [6]. Women were asked to fast on the day of their mammogram prior to providing a blood sample [6]. Blood samples were sent to the Tissue Procurement Core at WU Siteman Cancer Center within 30 min of collection and stored at −80 °C [18].

### Lipidomics profiling

Blood samples from each woman were sent to Metabolon (Durham, NC®) for comprehensive quantitative lipidomic profiling. Lipidomic profiling quantified 982 lipid species in 3 lipid super pathways and 14 sub-pathways: phospholipids (phosphatidylcholines (PC), lysophosphatidylcholines (LPC), phosphatidylethanolamines (PE), lysophosphatidylethanolamines (LPE), and phosphatidylinositols (PI)), sphingolipids (ceramides (CER), dihydroceramides (DCER), hexosylceramides (HCER), lactosylceramides (LCER), and sphingomyelins (SM)) and neutral complex lipids (cholesteryl esters (CE), diacylglycerols (DAG), triacylglycerols (TAG), and monoacylglycerols (MAG). Individual lipid species were quantified by taking the ratio of the signal intensity of each target compound to that of its assigned internal standard, then multiplying by the concentration of internal standard added to the sample [19]. Quality control samples, a large pool of human plasma maintained by Metabolon, were included in the assay runs and median relative standard deviation (RSD) percent values were calculated for each run. The overall median RSD across runs was 8%. Lipid sub-pathway concentrations were calculated from the sum of all molecular species within a sub-pathway [19].

### Mammographic breast density assessment

We assessed volumetric measures of MBD (volumetric percent density (%) (VPD), dense volume (cm^3^) (DV), and non-dense volume (cm^3^) (NDV)) using Volpara 1.5 (Volpara Health®). VPD was calculated by using the maximum fibroglandular volume between the left and right breasts. VPD was calculated by dividing DV by total breast volume and multiplying by 100. VPD was also categorized into: < 3.5%, 3.5% ~ 7.5%, > 7.5% ~ 15.5%, and > 15.5%, corresponding to the MBD groups of almost entirely fatty, scattered areas of fibroglandular density, heterogeneously dense, and extremely dense.

### Statistical *analysis*

Distributions of demographic, reproductive, and clinical factors were summarized by the four VPD categories into means and standard deviations for continuous variables and counts and percentages for categorical variables. One hundred twenty-five lipid species missing in 300 or more of the 705 samples were excluded from the analyses, leaving 857 lipid species. Lipid species with less than 300 missing samples were imputed using the 10-nearest neighbor methods using the R package *impute*.[20] We excluded 5 women with missing MBD measures, leaving 700 in the analytic sample. Spearman correlation coefficients were calculated across the lipid sub-pathways and lipid species with MBD measures.

We performed covariate-adjusted multivariable linear regression models fitting each MBD outcome with the concentration of lipid sub-pathways and lipid species, in quantitative scale and in quartiles, to determine their associations. MBD measures were log_10_ transformed for normality. In the quantitative scale, each lipid sub-pathway/species was standardized to have a zero mean and a unit standard deviation. The following covariates were accounted for in covariate-adjusted analyses: age (continuous), age at menarche (continuous), body shape at age 10 (based on Stunkard pictogram), body fat % (continuous), race (non-Hispanic white, non-Hispanic black, other), family history of breast cancer (yes, no), oral contraceptive use (never, less than 1 year, 1–4 years, 5–9 years, more than 10 years), alcohol consumption (never, < 1 drink/week, 1–2 drinks/week, 3–5 drinks/week, and 6 + drinks/week), and parity/age at first birth (nulliparous, 1–2 children & < 25 years, 1–2 children & 25–29 years, 1–2 children & ≥ 30 years, ≥ 3 children & < 25 years, ≥ 3 children & ≥ 25 years). Body fat % was used instead of body mass index (BMI) because it explained a slightly greater proportion of variation in VPD (*R*^2^ = 0.45) than BMI (*R*^2^ = 0.43). BMI and body fat % were highly correlated (r = 0.88). Some of the covariates included in the analyses had > 1% missingness, including body shape at age 10 (*N* = 40, 5.7%), and body fat % (*N* = 25, 3.5%); hence, missing values in all covariates were imputed using multivariate imputation by chain equations method via the R package *mice*.[21] We report the least square means (LSM) of VPD, DV, and NDV by quartile of lipid sub-pathway and lipid species. We calculated the p-value for trend in the adjusted LSM along quartiles by setting all values within each quartile to the median of that quartile range and operationalizing it as an ordinal variable. Linear coefficients were back-transformed. Residuals from the linear regression models were graphically examined for model goodness-of-fit. The proportional odds ratio model was applied to the four VPD categories with each lipid class as a predictor with the inclusion of the covariates to estimate the adjusted odds ratio with 95% CI. We corrected for multiple testing and the family-wise error rate using false discovery rate (FDR), and Bonferroni method, respectively.

To identify variables that influenced VPD the most, we bootstrapped the original dataset 200 times and applied least absolute shrinkage and selection operator (LASSO) penalized multivariable linear regression to each bootstrapped dataset to fit VPD with consideration of all available variables (the lipid sub-pathways, lipid species, and the covariates). We calculated the frequency of each variable being retained in the model fitted from the 200 bootstrapped datasets as an importance measure of the variables in predicting VPD. Model goodness-of-fit was evaluated based on the adjusted R^2^ value after sequentially adding lipid species to the model.

## Results

The mean age, BMI, and body fat % of study participants were 46 years, 30 kg/m^2^, and 40.4%, respectively; approximately 71.8% of participants were non-Hispanic white, and 23.1% were non-Hispanic black (Table 1). Almost half of the women in the study (44.9%) had scattered areas of fibroglandular density (VPD of 3.5% ~ 7.5%), and 28.1% had heterogeneously dense breasts.

**Table 1:** Characteristics of Premenopausal Women Recruited During Annual Screening Mammogram across Volumetric Percent Density Categories^a^

	Almost entirely fatty < 3.5% (*N* = 35)	Scattered areas of fibroglandular density 3.5% ~ 7.5% (*N* = 314)	Heterogeneously dense > 7.5% ~ 15.5% (*N* = 197)	Extremely dense > 15.5% (*N* = 154)	Overall (*N* = 705)
*Race*					
White	23 (65.7%)	203 (64.6%)	146 (74.1%)	130 (84.4%)	506 (71.8%)
Black	10 (28.6%)	94 (29.9%)	41 (20.8%)	17 (11.0%)	163 (23.1%)
Other	1 (2.9%)	15 (4.8%)	8 (4.1%)	7 (4.5%)	31 (4.4%)
Missing	1 (2.9%)	2 (0.6%)	2 (1.0%)	0 (0%)	5 (0.7%)
*Age, years*					
Mean (SD)	46.2 (5.4)	46.3 (4.4)	46.0 (4.3)	45.2 (4.5)	46.0 (4.5)
*Age at Menarche, years*					
Mean (SD)	12.5 (1.6)	12.4 (1.6)	12.8 (1.5)	13.0 (1.6)	12.7 (1.6)
Missing	1 (2.9%)	0 (0%)	1 (0.5%)	0 (0%)	2 (0.3%)
*Body Shape at Age 10*					
1&2	8 (22.9%)	95 (30.3%)	96 (48.7%)	88 (57.1%)	290 (41.1%)
3&4	15 (42.9%)	111 (35.4%)	62 (31.5%)	49 (31.8%)	237 (33.6%)
5	6 (17.1%)	44 (14.0%)	24 (12.2%)	11 (7.1%)	85 (12.1%)
6–9	6 (17.1%)	39 (12.4%)	6 (3.0%)	1 (0.6%)	53 (7.5%)
Missing	0 (0%)	25 (8.0%)	9 (4.6%)	5 (3.2%)	40 (5.7%)
*BMI, (kg/m*^*2*^*)*					
Mean (SD)	38.2 (6.8)	34.0 (6.9)	27.2 (4.9)	23.7 (3.7)	30.0 (7.5)
*Body Fat, (%)*					
Mean (SD)	49.4 (5.1)	45.6 (6.9)	37.9 (7.3)	31.3 (7.4)	40.4 (9.3)
Missing	4 (11.4%)	14 (4.5%)	3 (1.5%)	4 (2.6%)	25 (3.5%)
*Parity and Age at First Birth*					
Nulliparous	7 (20.0%)	67 (21.3%)	43 (21.8%)	43 (27.9%)	162 (23.0%)
1–2 children, < 25 years	6 (17.1%)	65 (20.7%)	24 (12.2%)	11 (7.1%)	107 (15.2%)
1–2 children,25–29 years	3 (8.6%)	44 (14.0%)	36 (18.3%)	27 (17.5%)	111 (15.7%)
1–2 children, ≥ 30 years	11 (31.4%)	53 (16.9%)	49 (24.9%)	43 (27.9%)	156 (22.1%)
≥ 3 children, < 25 years	3 (8.6%)	58 (18.5%)	15 (7.6%)	12 (7.8%)	88 (12.5%)
≥ 3 children, ≥ 25 years	5 (14.3%)	25 (8.0%)	30 (15.2%)	18 (11.7%)	79 (11.2%)
Missing	0 (0%)	2 (0.6%)	0 (0%)	0 (0%)	2 (0.3%)
*Family History of Breast Cancer*					
No	29 (82.9%)	251 (79.9%)	152 (77.2%)	104 (67.5%)	540 (76.6%)
Yes	5 (14.3%)	57 (18.2%)	44 (22.3%)	46 (29.9%)	152 (21.6%)
Missing	1 (2.9%)	6 (1.9%)	1 (0.5%)	4 (2.6%)	13 (1.8%)
*Oral Contraceptive Use, years*					
No	5 (14.3%)	42 (13.4%)	18 (9.1%)	16 (10.4%)	82 (11.6%)
< 1 year	2 (5.7%)	27 (8.6%)	13 (6.6%)	12 (7.8%)	55 (7.8%)
1–4 years	9 (25.7%)	66 (21.0%)	31 (15.7%)	35 (22.7%)	141 (20.0%)
5–9 years	5 (14.3%)	59 (18.8%)	47 (23.9%)	30 (19.5%)	143 (20.3%)
> 10 years	14 (40.0%)	119 (37.9%)	87 (44.2%)	60 (39.0%)	281 (39.9%)
Missing	0 (0%)	1 (0.3%)	1 (0.5%)	1 (0.6%)	3 (0.4%)
*Alcohol consumption, /per week*					
0 drinks	13 (37.1%)	107 (34.1%)	51 (25.9%)	39 (25.3%)	211 (29.9%)
< 1 drink	8 (22.9%)	85 (27.1%)	55 (27.9%)	32 (20.8%)	181 (25.7%)
1–2 drinks	9 (25.7%)	55 (17.5%)	37 (18.8%)	31 (20.1%)	133 (18.9%)
3–5 drinks	4 (11.4%)	46 (14.6%)	39 (19.8%)	37 (24.0%)	127 (18.0%)
6–10 drinks	0 (0%)	18 (5.7%)	12 (6.1%)	12 (7.8%)	43 (6.1%)
> 10 drinks	1 (2.9%)	3 (1.0%)	3 (1.5%)	2 (1.3%)	9 (1.3%)
Missing	0 (0%)	0 (0%)	0 (0%)	1 (0.6%)	1 (0.1%)
*VPD, (%)*					
Mean (SD)	3.15 (0.3)	5.16 (1.1)	10.9 (2.3)	22.6 (6.2)	10.5 (7.6)
*DV, (cm*^*3*^*)*					
Mean (SD)	57.7 (20.6)	72.3 (33.5)	81.7 (41.4)	108 (62.2)	82.0 (45.5)
*NDV, (cm*^*3*^*)*					
Mean (SD)	1870 (674)	1470 (690)	752 (392)	428 (258)	1060 (717)

a. 5 participants are missing VPD category, categories may not equal overall value

Abbreviations: standard deviation (SD), volumetric percent density (VPD), dense volume (DV), non-dense volume (NDV)

### Correlations between lipid sub-pathways/species and MBD

Lipid species in the TAG, LCER, LPC, and PC sub-pathways were most strongly correlated with VPD, NDV, and DV (Additional file 1: Fig. 1, and Additional file 2: Table 1). The strongest positive correlations observed with VPD were with LPC(18:1) (r = 0.39, p-value = 4.4E−27); with NDV was TAG54:6-FA20:4 (r = 0.51, p-value = 3.3E−47); and with DV was TAG58:10-FA20:4 (r = 0.14, p-value = 0.0003). The strongest inverse correlations for VPD were TAG54:6-FA20:4 (r = − 0.47, p−value = 8.6E−40); with NDV was LPC(18:1) (r = − 0.44, p-value = 1.9E−34); and with DV was PC(18:1/18:2) (r = −0.10, p-value = 0.008), (Additional file 2: Table 1).

### Multivariable linear regression between lipid sub-pathways/species and MBD

**Fig. 1:**
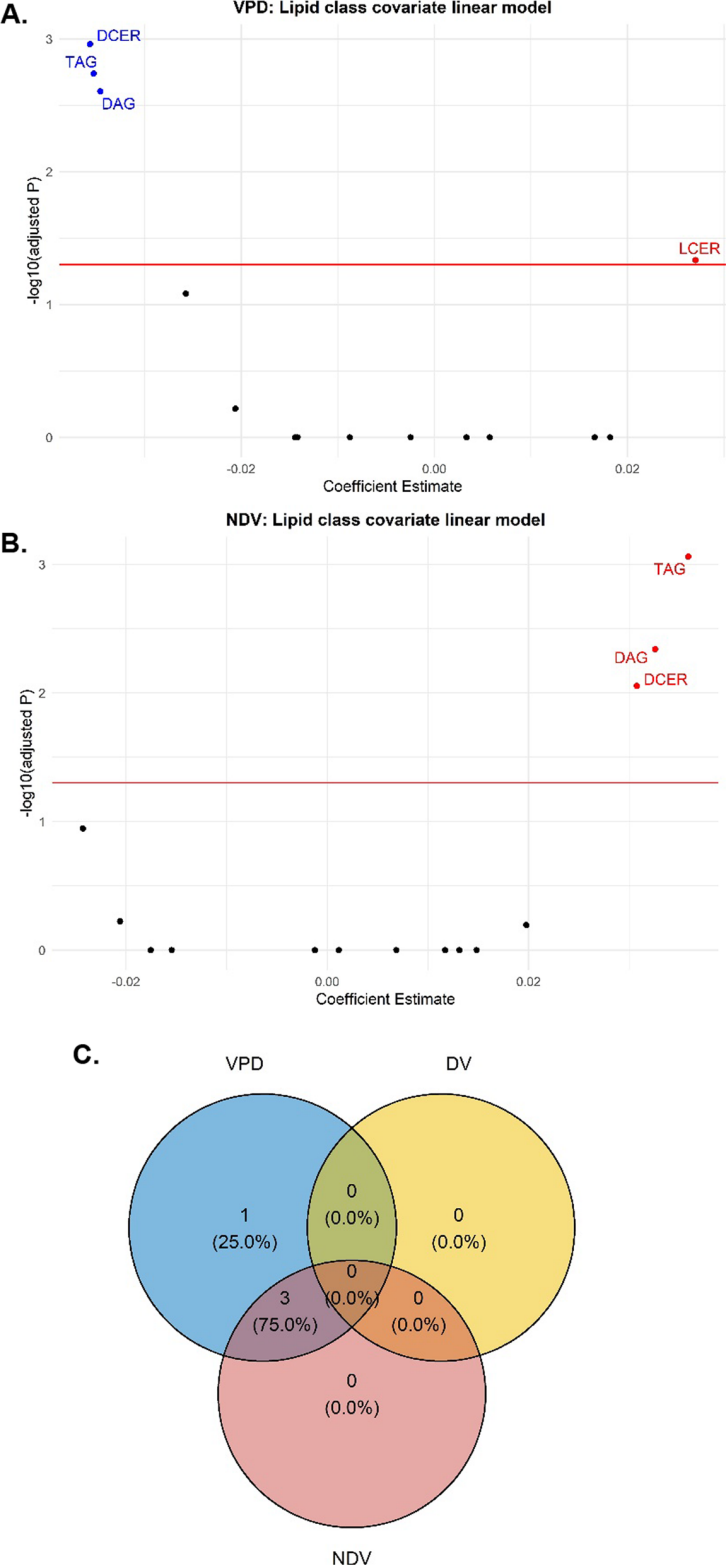
Covariate-adjusted Associations between Lipid Sub-pathways with VPD and NDV **A** volcano plot of lipid sub pathways for VPD, **B** volcano plot lipid sub-pathways for NDV, **C** Venn diagram of lipid sub-pathways for VPD, DV and NDV Abbreviations: volumetric percent density (VPD), non-dense volume (NDV) dihydroceramide (DCER), lactosylceramide (LCER), diacylglycerol (DAG), triacylglycerol (TAG)

**Fig. 2:**
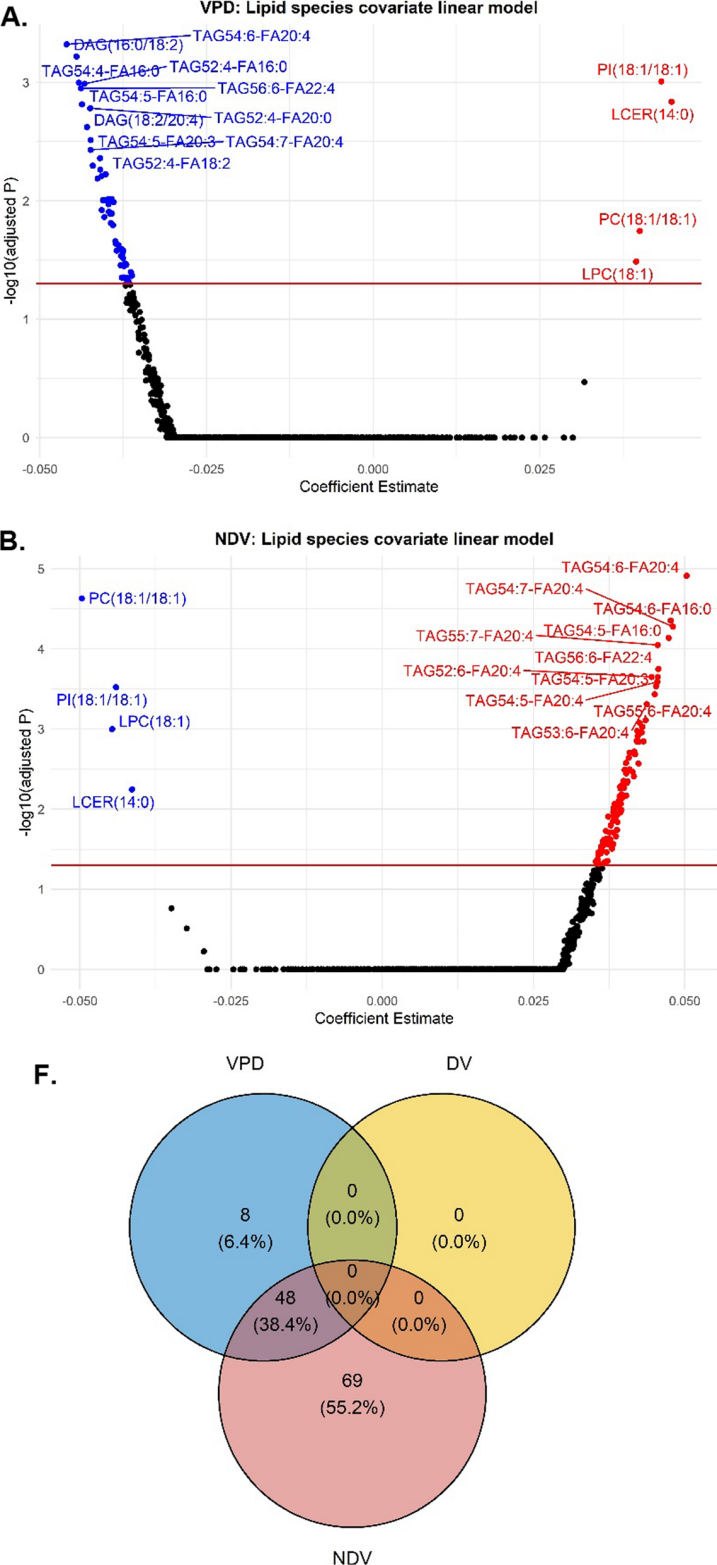
Covariate-adjusted Associations between Lipid Species with VPD and NDV **A** volcano plot of lipid species for VPD, **B** volcano plot of lipid species for NDV, **C** Venn diagram of lipid species for VPD, DV and NDV. Abbreviations: volumetric percent density (VPD), non-dense volume (NDV) lysophosphatidylcholine (LPC), phosphatidylinositol (PI), lactosylceramide (LCER), diacylglycerol (DAG), phosphatidylcholine (PC), triacylglycerol (TAG)

At the sub-pathway level, 4 of the 9 lipid sub-pathways (DCER, LCER, DAG, and TAG) were significantly associated with VPD (Bonferroni p value < 0.05) in the covariate-adjusted linear regression analyses (Fig. 1A and Additional file 1: Table 2). Fifty-six lipid species across seven lipid sub-pathways were significantly associated with VPD: TAG (N = 43), DAG (N = 7), PC (N = 2), PI (N = 1), LPC (N = 1), LCER (N = 1), and CE (N = 1). Four of the 56 lipid species were positively associated: LCER(14:0), LPC(18:1), PC(18:1/18:1), and PI(18:1/18:1), while the remaining 52 were inversely associated with VPD (Fig. 2A). Of the 52 lipid species that were inversely associated with VPD, 43 were from the TAG sub-pathway. The TAG species with similar chain lengths that were significantly associated with VPD displayed strong positive correlations ranging from 0.72–0.99 for TAG50 species, 0.58–0.99 for TAG52, and 0.69–0.97 for TAG54. One standard deviation increase in TAG54:6-FA20:4 was associated with a > 10% decrease in VPD, while one standard deviation increase in LCER(14:0) and PI(18:1/18:1) was associated with a > 10% increase in VPD. Three lipid sub-pathways (DCER, DAG, and TAG) were significantly associated with NDV (Fig. 1B and Additional file 1: Table 2). One hundred and seventeen lipid species were significantly associated with NDV, with 113 lipid species from the DAG (N = 14) and TAG (N = 99) sub-pathways showing positive associations, and 4 species (LCER(14:0), LPC(18:1), PC(18:1/18:1), and PI(18:1/18:1)) showing inverse associations (Fig. 2B). Three lipid sub-pathways and 48 lipid species (38.4%) were significantly associated with both VPD and NDV (Fig. 1C and Fig. 2C), and in opposite directions as expected. Of note, one standard deviation in TAG54:6-FA20:4 was associated with a > 10% change in both VPD (decrease) and NDV (increase). No lipid sub-pathway or species was significantly associated with DV.

### Multivariable covariate-adjusted least square means of volumetric percent density by quartiles of lipid sub-pathways/species

We categorized the lipid sub-pathways and species into quartiles and then calculated the least squared means (LSM) of VPD and NDV across the quartiles for those that were significant in the multiple linear regression analysis (Figs. 1&2). The four lipid sub-pathways, DCER, LCER, DAG, and TAG, were still associated with VPD after both FDR and Bonferroni correction (Table 2). All the 56 lipid species that were associated with VPD in continuous scale (Fig. 2A) were still significantly associated with VPD in quartiles at an FDR p-value < 0.05 and 48 at a more stringent Bonferroni p-value < 0.05 (Table 3). PI (18:1/18:1) was the lipid species with the strongest positive association with VPD. VPD increased across quartiles of PI(18:1/18:1): (Q1 = 7.5%, Q2 = 7.7%, Q3 = 8.4%, Q4 = 9.4%,FDR p-trend = 8.8E-05; Bonferroni p-trend = 0.02), (Table 3). All the 117 lipid species that were significantly associated with NDV in continuous scale (Fig. 2B) were still significantly associated with NDV at an FDR p-trend < 0.05, but only 101 were associated with NDV at a Bonferroni p-trend < 0.05, (Additional file 1: Table 3). Quartiles of DCER, LCER, DAG, and TAG were associated with NDV (Additional file 1: Table 4). There were no significant associations between lipid sub-pathways/lipid species and DV (Additional file 1: Table 4).

**Table 2:** Covariate-adjusted Least Square Means of Volumetric Percent Density by Quartiles of the 14 Lipid Sub-pathways

*Lipid pathway* ^a,b,c^	Q1LSM (95% CI)	Q2LSM (95% CI)	Q3LSM (95% CI)	Q4LSM (95% CI)	p-trend	FDR p-trend	Bonferroni p-trend
*Phospholipids*							
PC	8.5 (7.7–9.5)	8.4 (7.5–9.3)	7.9 (7.1–8.8)	8.1 (7.3–9.0)	0.30	0.55	1
LPC	7.6 (6.9–8.5)	8.4 (7.6–9.3)	8.4 (7.6–9.3)	8.5 (7.7–9.5)	0.07	0.18	1
PE	8.7 (7.8–9.6)	8.0 (7.2–8.9)	8.3 (7.5–9.3)	7.9 (7.1–8.8)	0.15	0.35	1
LPE	8.1 (7.3–9.0)	8.1 (7.3–9.0)	8.3 (7.5–9.2)	8.4 (7.6–9.3)	0.46	0.63	1
PI	8.3 (7.5–9.2)	8.4 (7.6–9.4)	8.2 (7.4–9.1)	8.0 (7.2–8.9)	0.43	0.63	1
*Sphingolipids*							
CER	9.0 (8.1–9.9)	8.4 (7.6–9.3)	7.6 (6.9–8.5)	7.8 (7.1–8.7)	0.008	0.04	0.33
**DCER**	**9.2 (8.3–10.2)**	**8.2 (7.4–9.1)**	**7.9 (7.1–8.7)**	**7.5 (6.8–8.4)**	**0.0004**	**0.003**	**0.02**
HCER	7.7 (6.9–8.5)	8.5 (7.6–9.4)	8.2 (7.4–9.1)	8.7 (7.8–9.6)	0.05	0.14	1
**LCER**	**7.5 (6.8–8.3)**	**8.3 (7.5–9.2)**	**8.2 (7.4–9.1)**	**9.3 (8.3–10.3)**	**0.0002**	**0.002**	**0.009**
SM	8.3 (7.5–9.2)	8.6 (7.8–9.6)	8.3 (7.5–9.2)	7.6 (6.8–8.4)	0.05	0.15	1
*Neutral complex lipids*							
CE	8.4 (7.6–9.3)	8.7 (7.8–9.6)	8.2 (7.4–9.1)	7.5 (6.7–8.3)	0.01	0.04	0.42
**DAG**	**9.0 (8.1–9.9)**	**8.5 (7.7–9.5)**	**8.1 (7.3–9.0)**	**7.3 (6.6–8.1)**	**0.0002**	**0.002**	**0.008**
**TAG**	**8.9 (8.0–9.9)**	**9.2 (8.3–10.2)**	**7.7 (7.0–8.5)**	**7.2 (6.5–8.1)**	**1.7E-05**	**0.0003**	**0.0007**
MAG	8.6 (7.7–9.5)	8.1 (7.3–8.9)	7.9 (7.1–8.7)	8.4 (7.6–9.4)	0.63	0.71	1

^a^Models were adjusted for age (continuous), age at menarche (continuous), body fat % (continuous), race (non-Hispanic white, non-Hispanic black,

other), family history of breast cancer (yes, no), oral contraceptive use (never, less than 1 year, 1–4 years, 5–9 years, more than 10 years),

alcohol consumption (never, less than 1 drink per week, 1–2 drinks per week, 3–5 drinks per week, and 6 + drinks per week), parity/age at first birth (nulliparous, 1–2 children & < 25 years, 1–2 children & 25–29 years, 1–2 children &, ≥ 30 years, ≥ 3 children & < 25 years,

≥ 3 children & ≥ 25 years) and body shape at age 10 (1&2, 3&4, 5, 6–9)

^b^Volumetric percent density was log_10_ transformed and coefficients were back-transformed (10^β), and bolded if the Bonferroni p-trend is < 0.05

^c^Quartile values: PC, Q1 = (1165 μM-1848 μM), Q2 = (> 1848 μM-2097 μM), Q3 = (> 2097 μM-2460 μM), Q4 = (> 2460 μM-4298 μM); LPC, Q1 = (60.1 μM-144 μM),

Q2 = (> 144 μM-169 μM), Q3 = (> 169 μM-199 μM), Q4 = (> 199 μM-312 μM); PE, Q1 = (76.6-167 μM), Q2 = (> 167-195 μM), Q3 = (> 195-231 μM), Q4 = (> 231-471 μM);

LPE, Q1 = (2.34–4.82 μM), Q2 = (> 4.82–5.84 μM), Q3 = (> 5.84–7.08 μM), Q4 = (> 7.08–12.04 μM); PI, Q1 = (2.68,-6.44 μM), Q2 = (> 6.44–7.75 μM),

Q3 = (> 7.75–9.39 μM), Q4 = (> 9.39–18.77 μM); CER, Q1 = (3.04–5.58 μM), Q2 = (> 5.58–6.49 μM), Q3 = (> 6.49–7.43 μM), Q4 = (> 7.43–15.57 μM); DCER, Q1 = (0.879–1.66 μM), Q2 = (> 1.66–1.92 μM), Q3 = (> 1.92–2.25 μM), Q4 = (> 2.25–4.60 μM); HCER, Q1 = (1.78–3.38 μM), Q2 = (> 3.38–4.02 μM), Q3 = (> 4.02–4.67 μM), Q4 = (> 4.67–10.27 μM); LCER, Q1 = (1.47–2.92 μM), Q2 = (> 2.92–3.56 μM), Q3 = (> 3.56–4.29 μM), Q4 = (> 4.29–17.27 μM); SM, Q1 = (354-523 μM), Q2 = (> 523-586 μM), Q3 = (> 586-643 μM), Q4 = (> 643-1118 μM); CE, Q1 = (1066-1832 μM), Q2 = (> 1832-2062 μM), Q3 = (> 2062-2337 μM), Q4 = (> 2337-4335 μM); DAG, Q1 = (5.26–16.1 μM), Q2 = (> 16.1–22.1 μM), Q3 = (> 22.1–32.3 μM), Q4 = (> 32.3–106.8 μM); TAG, Q1 = (276-748 μM), Q2 = (> 748-1018 μM), Q3 = (> 1018-1516 μM), Q4 = (> 1516-6296 μM); MAG, Q1 = (0.23–0.79 μM), Q2 = (> 0.79–1.71 μM), Q3 = (> 1.71–12.89 μM), Q4 = (> 12.89–56.82 μM); Abbreviations: phosphatidylcholine (PC), lysophosphatidylcholine (LPC), phosphatidylethanolamine (PE), lysophosphatidylethanolamine (LPE),

phosphatidylinositol (PI), ceramide (CER), dihydroceramide (DCER), hexosylceramide (HCER), lactosylceramide (LCER), sphingomyelin (SM),

cholesteryl ester (CE), diacylglycerol (DAG), triacylglycerol (TAG), monoacylglycerol (MAG), quartiles 1–4 (Q1-4), least square mean (LSM),

confidence interval (CI), false discovery rate (FDR)

**Table 3:** Covariate-adjusted Least Square Means of Volumetric Percent Density (VPD) by Quartiles of Lipid Species that were Significantly Associated with VPD at a Bonferroni P-value < 0.05 ^a,b,c^

	Q1 LSM (95% CI)	Q2 LSM (95% CI)	Q3 LSM (95% CI)	Q4 LSM (95% CI)	p-trend	FDR p-trend	Bonferroni p-trend
*Phospholipids*							
**PC(18:0/20:3)**	**9.0 (8.2–10.0)**	**8.4 (7.6–9.3)**	**8.1 (7.3–9.0)**	**7.1 (6.4–7.9)**	**1.2E-05**	**0.0001**	**0.03**
**PC(18:1/18:1)**	**7.4 (6.7–8.2)**	**8.2 (7.4–9.0)**	**8.3 (7.4–9.2)**	**9.5 (8.6–10.6)**	**1.1E-05**	**0.0001**	**0.03**
LPC(18:1)	7.4 (6.6–8.2)	7.7 (7.0–8.5)	9.1 (8.2–10.1)	8.8 (7.9–9.8)	0.0006	0.002	1
**PI(18:1/18:1)**	**7.5 (6.7–8.3)**	**7.7 (7.0–8.6)**	**8.4 (7.6–9.3)**	**9.4 (8.5–10.5)**	**6.3E-06**	**8.8E-05**	**0.02**
*Sphingolipids*							
**LCER(14:0)**	**7.4 (6.7–8.2)**	**8.2 (7.4–9.0)**	**8.6 (7.8–9.6)**	**9.6 (8.6–10.7)**	**1.2E-05**	**0.0001**	**0.03**
*Neutral complex lipids*							
**CE(20:3)**	**9.3 (8.4–10.3)**	**8.2 (7.4–9.0)**	**7.7 (6.9–8.5)**	**7.1 (6.4–8.0)**	**2.4E-06**	**5.0E-05**	**0.006**
**DAG(14:0/18:2)**	**8.8 (8.0–9.8)**	**8.7 (7.8–9.6)**	**8.2 (7.4–9.1)**	**7.1 (6.4–7.9)**	**7.9E-06**	**0.0001**	**0.02**
**DAG(16:0/18:2)**	**9.4 (8.5–10.5)**	**9.1 (8.2–10.1)**	**7.4 (6.7–8.2)**	**7.2 (6.5–8.0)**	**6.5E-07**	**2.7E-05**	**0.002**
**DAG(16:0/18:3)**	**9.2 (8.3–10.2)**	**9.1 (8.2–10.1)**	**7.6 (6.8–8.4)**	**7.2 (6.5–8.0)**	**2.7E-06**	**5.2E-05**	**0.007**
**DAG(16:1/18:2)**	**9.3 (8.3–10.3)**	**8.7 (7.8–9.6)**	**7.7 (6.9–8.5)**	**7.0 (6.3–7.9)**	**2.6E-06**	**5.2E-05**	**0.007**
DAG(18:0/18:2)	9.1 (8.2–10.2)	8.9 (8.1–9.9)	7.6 (6.8–8.4)	7.3 (6.6–8.1)	2.6E-05	0.0002	0.07
DAG(18:2/20:4)	9.4 (8.5–10.4)	8.3 (7.5–9.2)	8.0 (7.2–8.9)	7.3 (6.5–8.1)	2.3E-05	0.0002	0.06
DAG(18:2/22:4)	9.0 (8.1–10.0)	8.4 (7.6–9.3)	7.8 (7.0–8.7)	7.2 (6.4–8.0)	3.0E-05	0.0002	0.08
**TAG50:2-FA16:0**	**9.2 (8.3–10.2)**	**9.1 (8.2–10.0)**	**7.7 (7.0–8.6)**	**7.0 (6.3–7.8)**	**1.5E-07**	**1.2E-05**	**0.004**
**TAG50:2-FA18:2**	**9.3 (8.4–10.3)**	**8.9 (8.0–9.9)**	**7.9 (7.1–8.7)**	**7.1 (6.4–7.9)**	**3.2E-07**	**1.8E-05**	**0.0008**
**TAG50:3-FA16:0**	**9.4 (8.5–10.4)**	**9.2 (8.3–10.1)**	**7.5 (6.8–8.3)**	**6.9 (6.2–7.7)**	**1.2E-08**	**3.4E-06**	**3.1E-05**
**TAG50:3-FA18:2**	**9.1 (8.2–10.1)**	**9.0 (8.2–10.0)**	**7.6 (6.8–8.4)**	**7.1 (6.3–7.9)**	**1.3E-06**	**3.7E-05**	**0.003**
**TAG50:3-FA18:3**	**8.8 (8.0–9.8)**	**9.3 (8.4–10.3)**	**8.0 (7.2–8.8)**	**7.2 (6.5–7.9)**	**3.6E-06**	**6.1E-05**	**0.009**
**TAG50:4-FA14:0**	**9.0 (8.1–10.0)**	**8.2 (7.4–9.1)**	**8.2 (7.4–9.1)**	**7.1 (6.4–7.9)**	**1.6E-05**	**0.0002**	**0.04**
**TAG50:4-FA18:2**	**9.0 (8.1–9.9)**	**8.4 (7.6–9.3)**	**8.1 (7.3–9.0)**	**7.1 (6.3–7.9)**	**1.1E-05**	**0.0001**	**0.03**
**TAG51:3-FA16:0**	**9.4 (8.4–10.4)**	**8.6 (7.7–9.5)**	**7.8 (7.1–8.7)**	**7.1 (6.4–7.9)**	**1.9E-06**	**4.6E-05**	**0.005**
**TAG52:2-FA18:2**	**9.3 (8.4–10.4)**	**8.8 (7.9–9.7)**	**7.6 (6.8–8.4)**	**7.3 (6.6–8.1)**	**1.4E-05**	**0.0001**	**0.04**
**TAG52:2-FA20:2**	**9.0 (8.1–10.0)**	**8.7 (7.9–9.6)**	**8.5 (7.6–9.4)**	**6.9 (6.3–7.7)**	**5.3E-07**	**2.4E-05**	**0.001**
**TAG52:3-FA16:0**	**9.4 (8.5–10.5)**	**8.5 (7.7–9.4)**	**7.8 (7.0–8.7)**	**7.3 (6.5–8.1)**	**7.6E-06**	**9.9E-05**	**0.02**
**TAG52:3-FA18:1**	**9.4 (8.4–10.4)**	**8.5 (7.7–9.4)**	**7.9 (7.1–8.7)**	**7.2 (6.5–8.0)**	**3.7E-06**	**6.2E-05**	**0.009**
**TAG52:3-FA18:2**	**9.4 (8.5–10.4)**	**8.5 (7.7–9.4)**	**7.8 (7.1–8.7)**	**7.2 (6.5–8.0)**	**4.0E-06**	**6.5E-05**	**0.01**
**TAG52:3-FA20:0**	**9.5 (8.5–10.5)**	**8.6 (7.8–9.5)**	**7.8 (7.0–8.6)**	**7.1 (6.4–7.9)**	**5.4E-07**	**2.4E-05**	**0.001**
**TAG52:3-FA20:3**	**8.9 (8.0–9.8)**	**9.1 (8.2–10.0)**	**8.1 (7.3–9.0)**	**7.0 (6.3–7.8)**	**7.0E-07**	**2.7E-05**	**0.002**
**TAG52:3-FA22:1**	**9.2 (8.3–10.2)**	**8.8 (7.9–9.7)**	**7.9 (7.1–8.8)**	**7.1 (6.4–7.9)**	**1.9E-06**	**4.5E-05**	**0.005**
**TAG52:4-FA14:0**	**9.0 (8.1–9.9)**	**8.4 (7.6–9.3)**	**8.3 (7.5–9.2)**	**6.9 (6.2–7.7)**	**1.3E-06**	**3.6E-05**	**0.003**
**TAG52:4-FA16:0**	**9.6 (8.7–10.6)**	**8.8 (8.0–9.8)**	**7.4 (6.7–8.2)**	**7.3 (6.6–8.1)**	**4.7E-07**	**2.3E-05**	**0.001**
**TAG52:4-FA18:2**	**9.5 (8.6–10.5)**	**8.7 (7.8–9.6)**	**7.6 (6.9–8.4)**	**7.2 (6.5–8.0)**	**3.8E-07**	**2.0E-05**	**0.001**
**TAG52:4-FA20:0**	**9.6 (8.6–10.6)**	**8.8 (8.0–9.8)**	**7.4 (6.7–8.2)**	**7.3 (6.6–8.1)**	**5.3E-07**	**2.4E-05**	**0.001**
TAG52:4-FA20:2	9.2 (8.3–10.2)	8.4 (7.5–9.3)	7.8 (7.0–8.6)	7.3 (6.5–8.1)	5.2E-05	0.0004	0.13
**TAG52:4-FA22:1**	**9.4 (8.5–10.5)**	**8.6 (7.8–9.6)**	**7.6 (6.8–8.4)**	**7.3 (6.6–8.1)**	**4.1E-06**	**6.6E-05**	**0.01**
**TAG52:5-FA16:0**	**9.4 (8.5–10.4)**	**8.9 (8.0–9.9)**	**7.6 (6.9–8.5)**	**7.2 (6.5–8.0)**	**1.1E-06**	**3.3E-05**	**0.003**
**TAG52:5-FA20:3**	**9.2 (8.4–10.2)**	**8.1 (7.3–9.0)**	**8.1 (7.3–9.1)**	**7.0 (6.3–7.8)**	**2.4E-06**	**5.0E-05**	**0.006**
**TAG52:6-FA20:4**	**9.0 (8.1–9.9)**	**8.9 (8.1–9.9)**	**7.8 (7.0–8.6)**	**7.2 (6.5–8.0)**	**1.5E-05**	**0.0002**	**0.04**
**TAG53:4-FA16:0**	**9.5 (8.5–10.5)**	**8.4 (7.6–9.3)**	**7.9 (7.2–8.8)**	**7.2 (6.5–8.0)**	**4.5E-06**	**7.0E-05**	**0.01**
TAG54:3-FA16:0	8.9 (8.0–9.9)	8.7 (7.8–9.6)	7.9 (7.1–8.8)	7.1 (6.4–7.9)	2.1E-05	0.0002	0.05
**TAG54:4-FA16:0**	**9.1 (8.2–10.1)**	**8.8 (8.0–9.8)**	**7.6 (6.9–8.5)**	**7.1 (6.4–7.9)**	**2.8E-06**	**5.3E-05**	**0.007**
TAG54:4-FA20:2	9.2 (8.3–10.2)	8.2 (7.4–9.1)	8.1 (7.3–8.9)	7.3 (6.5–8.1)	6.3E-05	0.0004	0.16
**TAG54:4-FA20:3**	**9.0 (8.1–10.0)**	**8.8 (8.0–9.7)**	**7.9 (7.1–8.8)**	**7.1 (6.4–7.9)**	**5.5E-06**	**8.1E-05**	**0.01**
**TAG54:5-FA16:0**	**9.4 (8.4–10.4)**	**8.9 (8.1–9.9)**	**7.7 (6.9–8.6)**	**6.9 (6.2–7.7)**	**6.4E-08**	**7.5E-06**	**0.0002**
TAG54:5-FA20:2	9.1 (8.2–10.1)	8.4 (7.6–9.3)	7.8 (7.0–8.7)	7.4 (6.6–8.2)	0.0003	0.001	0.71
**TAG54:5-FA20:3**	**9.5 (8.6–10.5)**	**8.4 (7.6–9.3)**	**7.8 (7.0–8.6)**	**7.0 (6.3–7.8)**	**2.9E-07**	**1.7E-05**	**0.0007**
**TAG54:5-FA20:4**	**9.5 (8.5–10.5)**	**8.8 (7.9–9.7)**	**7.6 (6.9–8.5)**	**7.2 (6.5–8.0)**	**6.6E-06**	**9.0E-05**	**0.02**
**TAG54:6-FA16:0**	**9.7 (8.7–10.7)**	**8.7 (7.8–9.6)**	**7.9 (7.1–8.7)**	**7.0 (6.3–7.7)**	**2.6E-08**	**5.2E-06**	**6.8E-05**
**TAG54:6-FA20:3**	**9.4 (8.5–10.4)**	**8.3 (7.5–9.2)**	**7.7 (6.9–8.5)**	**7.2 (6.4–8.0)**	**3.7E-06**	**6.2E-05**	**0.01**
**TAG54:6-FA20:4**	**9.8 (8.8–10.9)**	**8.6 (7.8–9.6)**	**7.6 (6.8–8.4)**	**7.0 (6.3–7.8)**	**8.1E-08**	**8.7E-06**	**0.0002**
**TAG54:7-FA20:4**	**9.5 (8.6–10.5)**	**8.9 (8.0–9.8)**	**7.4 (6.7–8.3)**	**6.9 (6.2–7.7)**	**4.4E-08**	**6.5E-06**	**0.0001**
**TAG55:7-FA20:4**	**9.2 (8.2–10.2)**	**8.8 (8.0–9.8)**	**7.7 (7.0–8.6)**	**7.2 (6.5–8.1)**	**3.9E-06**	**6.4E-05**	**0.01**
**TAG56:4-FA22:4**	**9.1 (8.2–10.1)**	**9.0 (8.2–10.0)**	**7.7 (6.9–8.5)**	**7.1 (6.4–7.9)**	**2.0E-06**	**4.7E-05**	**0.005**
**TAG56:5-FA22:4**	**9.1 (8.2–10.2)**	**8.7 (7.8–9.6)**	**8.0 (7.2–8.9)**	**7.2 (6.5–8.0)**	**1.4E-05**	**0.0001**	**0.04**
**TAG56:6-FA22:4**	**9.6 (8.7–10.7)**	**8.6 (7.8–9.5)**	**7.6 (6.9–8.5)**	**7.1 (6.4–7.9)**	**1.9E-07**	**1.3E-05**	**0.0005**
**TAG56:7-FA22:4**	**9.3 (8.4–10.3)**	**8.9 (8.0–9.8)**	**7.5 (6.7–8.3)**	**7.3 (6.5–8.1)**	**7.0E-06**	**9.4E-05**	**0.02**

^a^Models were adjusted for age (continuous), age at menarche (continuous), body fat % (continuous), race (non-Hispanic white, non-Hispanic black, other), family history of breast cancer (yes, no), oral contraceptive use (never, less than 1 year, 1–4 years, 5–9 years, more than 10 years), alcohol consumption (never, less than 1 drink per week, 1–2 drinks per week, 3–5 drinks per week, and 6 + drinks per week), parity/age at first birth (nulliparous, 1–2 children & < 25 years, 1–2 children & 25–29 years, 1–2 children &, ≥ 30 years, ≥ 3 children & < 25 years, ≥ 3 children & ≥ 25 years) and body shape at age 10 (1&2, 3&4, 5, 6–9)

^b^Volumetric percent density was log_10_ transformed and coefficients were back-transformed (10^β)

Abbreviations: phosphatidylcholine (PC), lysophosphatidylcholine (LPC), phosphatidylinositol (PI), lactosylceramide (LCER), cholesteryl ester (CE), diacylglycerol (DAG), triacylglycerol (TAG), quartiles 1–4 (Q1-4), least square mean (LSM), confidence interval (CI), false discovery rate (FDR)

^c^Lipid species presented were associated with volumetric percent density in multivariable linear regression analyses, after Bonferroni correction and bolded if the Bonferroni p-trend is < 0.05

### Proportional odds model

Additional file 1: Fig. 2 presents the results from the covariate-adjusted proportional odds model investigating the associations between lipid sub-pathways and the four VPD categories. LPC and LCER were significantly positively associated with VPD categories, while DCER, DAG, and TAG were inversely associated.

### LASSO regression and bootstrapping

Body fat %, body shape at age 10, parity/age at first birth, and family history of breast cancer were most strongly associated with VPD and were retained in the final lasso-penalized multivariable linear regression model in 100% of the 200 bootstrapped datasets. Some lipid species (e.g., PI(18:1/18:1)—99%, LCER (14:0), and PE(O-16:0/22:6)—98.5%) were selected at higher frequencies than classic covariates, such as age, race and age at menarche, that are associated with VPD. For VPD, the adjusted R^2^ (a model goodness-of-fit measure) derived from the model containing only the classic covariates was 0.45. A reasonably selected (based on the scree plot of the adjusted R^2^) model, which included all the covariates and 57 lipid species, rendered an improved adjusted *R*^2^ = 0.59 (results not shown).

## Discussion

Using untargeted comprehensive lipidomics profiling, we demonstrate, for the first time, the associations of lipid species with MBD in premenopausal women. We observed 56 lipid species that were associated with VPD and 117 lipid species with NDV. Most of these lipid species were from the large TAG sub-pathway. Some of the lipid species explain greater variation in VPD than well-established determinants of MBD and appear to improve model goodness-of-fit based on the adjusted R^2^ as the covariate-only model rendered an adjusted *R*^2^ = 0.45 while additionally including 57 lipid species increased the adjusted *R*^2^ to 0.59.

To the best of our knowledge, this is the first study to characterize the lipidome of MBD. Several lipid species were associated with MBD after adjustment for adiposity. Studies have investigated the associations of biomarkers of adiposity, such as adipokines and insulin-like growth factors with, MBD, but the findings are not consistent. The association of most of these biomarkers with MBD, particularly the adipokines, is influenced by BMI. Several studies found a null association between these biomarkers and MBD after adjusting for measures of adiposity [10, 11, 22–25], although some studies reported an inverse association between leptin and percent density [10, 11, 26]. The association of C-peptide with MBD is similarly attenuated once adjusted for BMI [9, 27]. Similarly, the associations of insulin-like growth factors with MBD are not consistent [12, 28–31]. Our study, therefore, provides important novel insights on the biological mechanisms underlying MBD.

Although no population-based studies have comprehensively characterized the lipidome of breast cancer, preclinical studies suggest that breast cancer subtypes have unique lipidomic profiles [32–34], and a few studies have evaluated the utility of a limited set of lipid species for their diagnostic performance in breast cancer development. One study profiled 110 lipid species in 121 breast cancer cases and 45 healthy controls;19 lipid species distinguished women with triple-negative breast cancer (TNBC) from non-cancer controls, and 5 lipid species distinguished women with TNBC from women with other types of breast cancer [35]. Another study compared lipid profiles in breast tissue of 42 women with breast cancer to 19 healthy controls, reporting 48 significantly differential lipid species. They identified higher levels of lipid species in the LPC sub-pathway but lower levels of lipids in ceramides, DAG, PC, and PE sub-pathways in breast tissues of women with breast cancer compared to healthy controls [36]. They found higher levels of LPC(18:1) in cancer tissue compared to normal tissue [36]. We found a significant positive association between LPC(18:1) and VPD; hence, LPC(18:1) may be a novel biomarker of VPD and breast cancer development. LPC(18:1) has been found to be abundant in MCF-7 cell lines (ER + /PR +) compared to MCF10A cell lines (normal) [32].

Lipids are generated in cells through de novo synthesis or by intake through exogenous sources [15]. Lipid species are a diverse group of compounds and are categorized by their primary function, which is determined by their polar head and are further differentiated by the addition of a hydrocarbon chain [37]. Lipids are functionally important in maintaining cell membranes and fueling the cell [15]. Lipids within the phospholipid and sphingolipid super pathways are a key component in cell membrane development, while neutral complex lipids, particularly DAG and TAG provide energy to fuel the cell [34].

Three of the 4 lipid species (PC(18:1/18:1), LPC(18:1), PI(18:1/18:1)) that were positively associated with VPD were from the phospholipid super pathway and the associations are biologically plausible. They contain at least one monounsaturated fatty acid (MUFA) chain. Higher levels of MUFAs have been reported in breast tumor tissues compared to normal tissues from the same woman [38], and some, but not others, have suggested associations of MUFAs with breast cancer risk [39]. PI(18:1/18:1) promotes cell survival and is responsive to diet and stress [40]. LPCs have an impact on innate immunity and have been shown to possess both anti-and pro-inflammatory properties [41]. Two studies exploring the relationship between circulating triglycerides and MBD observed null or inverse findings.[42, 43]. Our findings on the inverse associations of several TAG species with VPD, therefore, provide new information on triglycerides and MBD. Because of the very strong correlations of TAG species with the same chain lengths, studies are needed to determine which of the TAG species are more amenable to targeting breast cancer prevention.

### Strengths/limitations

Our study population is large and well annotated, with detailed information on demographic, reproductive, and anthropometric measures. This allowed us to perform robust analyses with control for confounders. The study population is diverse and mirrors the underlying population attending screening mammogram at our institution; hence, our results are generalizable to the source population which our study participants were recruited from or other populations of similar characteristics [8]. We performed lipidomic profiling on fasting blood samples, which should provide a more reliable measure of the lipid species than using non-fasted samples [44].

There are also some limitations. The study is cross-sectional; therefore, we cannot establish longitudinal trajectories of lipids. Due to the large number of lipid species evaluated, some findings may be due to chance. Nevertheless, we applied the stringent Bonferonni correction; hence, the potential of chance findings are considerably reduced. Our study is limited to premenopausal women; therefore, our results cannot be generalized to postmenopausal women.

## Conclusion

Our study offers new insights into the biological mechanisms underlying high MBD in premenopausal women. Additional studies are needed to validate our findings.

### Supplementary Information

**Additional file 1. Supplementary Figure 1.** Spearman Correlation Heatmap Plot across Lipid Sub-pathways and Mammographic Breast Density Measures. **Supplementary Table 2.** Multivariable Covariate-adjusted Linear Regression between Lipid Sub-pathways and Mammographic Breast Density Measures. **Supplementary Table 3.** Covariate-adjusted Least Square Means of Non-dense Volume (NDV) by Quartiles of Lipid Species that were Significantly Associated with NDV at a Bonferroni P-value<0.05. **Supplementary Table 4:** Covariate-adjusted Least Square Mean of Dense Volume and Non-dense Volume by Quartile of Lipid Sub-pathways. **Supplementary Figure 2:** Covariate-adjusted Proportional Odds Model between the 14 Lipid Sub-pathways and Volumetric Percent Density Categories.

**Additional file 2. Supplementary Table 1.** Spearman Correlations across Lipid Species and Mammographic Breast Density Measures (Excel file).

## Data Availability

De-identified data are available from the corresponding author upon reasonable request.

## Abbreviations

MBD: Mammographic breast density
WU: Washington university school of medicine
PC: Phosphatidylcholines
LPC: Lysophosphatidylcholines
PE: Phosphatidylethanolamines
LPE: Lysophosphatidylethanolamines
PI: Phosphatidylinositols
CER: Ceramides
DCER: Dihydroceramides
HCER: Hexosylceramides
LCER: Lactosylceramides
SM: Sphingomyelins
CE: Cholesteryl esters
DAG: Diacylglycerols
TAG: Triacylglycerols
MAG: Monoacylglycerols
VPD: Volumetric percent density
DV: Dense volume
NDV: Non-dense volume
BMI: Body mass index
LSM: Least square means
FDR: False discovery rate
LASSO: Least absolute shrinkage and selection operator
TNBC: Triple-negative breast cancer
MUFA: Monounsaturated fatty acid
SD: Standard deviation
CI: Confidence interval
Q1-4: Quartiles 1–4
RSD: Relative standard deviation
