# Supplementary material for: Lipidome of mammographic breast density in premenopausal women
Source: Breast Cancer Res. 2023 Oct 9;25:121. doi: 10.1186/s13058-023-01725-1 (PMC10561435; doi:10.1186/s13058-023-01725-1)
Supplement: Supplementary file 1 — Additional file 1. Supplementary Figure 1. Spearman Correlation Heatmap Plot across Lipid Sub-pathways and Mammographic Breast Density Measures. Supplementary Table 2. Multivariable Covariate-adjusted Linear Regression between Lipid Sub-pathways and Mammographic Breast Density Measures. Supplementary Table 3. Covariate-adjusted Least Square Means of Non-dense Volume (NDV) by Quartiles of Lipid Species that were Significantly Associated with NDV at a Bonferroni P-value<0.05. Supplementary Table 4: Covariate-adjusted Least Square Mean of Dense Volume and Non-dense Volume by Quartile of Lipid Sub-pathways. Supplementary Figure 2: Covariate-adjusted Proportional Odds Model between the 14 Lipid Sub-pathways and Volumetric Percent Density Categories. [file 13058_2023_1725_MOESM1_ESM.docx]

**Supplemental Tables and Figures**

**Supplementary Table 1.** Spearman Correlations across Lipid Species and Mammographic Breast Density Measures (Excel file)

**Supplementary Figure 1.** Spearman Correlation Heatmap Plot across Lipid Sub-pathways and Mammographic Breast Density Measures

**Supplementary Table 2.** Multivariable Covariate-adjusted Linear Regression between Lipid Sub-pathways and Mammographic Breast Density Measures

**Supplementary Table 3.** Covariate-adjusted Least Square Means of Non-dense Volume (NDV) by Quartiles of Lipid Species that were Significantly Associated with NDV at a Bonferroni P-value<0.05

**Supplementary Table 4:** Covariate-adjusted Least Square Mean of Dense Volume and Non-dense Volume by Quartile of Lipid Sub-pathways

**Supplementary Figure 2:** Covariate-adjusted Proportional Odds Model between the 14 Lipid Sub-pathways and Volumetric Percent Density Categories

**Supplementary Figure 1: Spearman Correlation Heatmap Plot across Lipid Sub-pathways and Mammographic Breast Density Measures^a^**


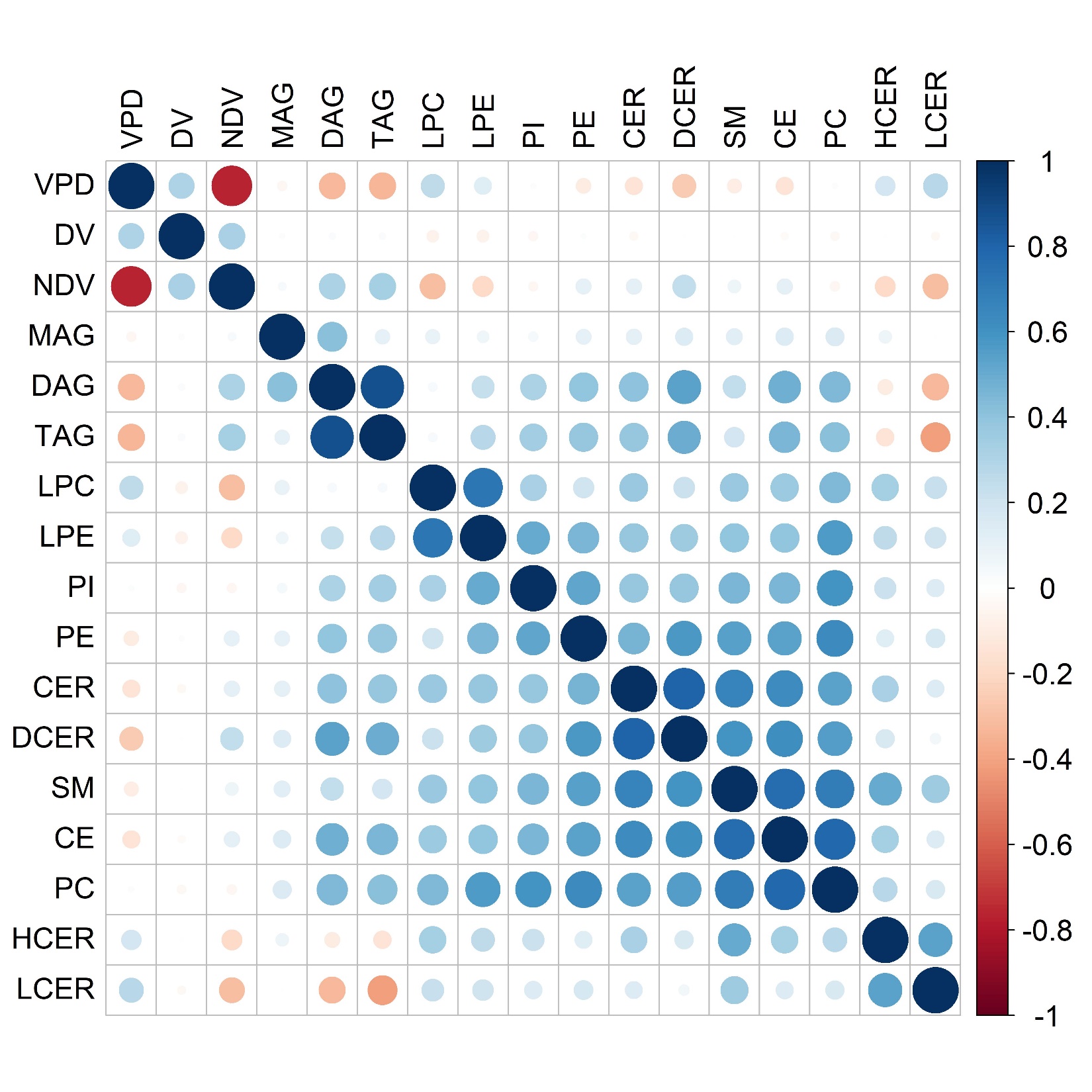


a. Only statistically significant correlations were shown in color while non-significant ones are blank. Abbreviations: volumetric percent density (VPD), dense volume (DV), non-dense volume (NDV), phosphatidylcholine (PC), lysophosphatidylcholine (LPC), phosphatidylethanolamine (PE), lysophosphatidylethanolamine (LPE), phosphatidylinositol (PI), ceramide (CER), dihydroceramide (DCER), hexosylceramide (HCER), lactosylceramide (LCER), sphingomyelin (SM), cholesteryl ester (CE), diacylglycerol (DAG), triacylglycerol (TAG), monoacylglycerol (MAG)

| **Supplementary Table 2: Multivariable Covariate-adjusted Linear Regression between Lipid Sub-pathways and Mammographic Breast Density Measures ^a,b^** | | | | | | | | | | | | | |
| --- | --- | --- | --- | --- | --- | --- | --- | --- | --- | --- | --- | --- | --- |
|  | | Volumetric Percent Density | | | | Non-dense Volume (cm^3^) | | | | Dense Volume (cm^3^) | | | |
|  | 10^(β) (95%CI) | | p-value | FDR  p-value | Bonferroni  p-value | 10^(β) (95%CI) | p-value | FDR  p-value | Bonferroni  p-value | 10^ (β) (95%CI) | p-value | FDR  p-value | Bonferroni  p-value |
| PC | 0.98 (0.94, 1.02) | | 0.31 | 0.52 | 1 | 1.02 (0.98, 1.06) | 0.42 | 0.62 | 1 | 0.99 (0.96, 1.03) | 0.79 | 0.87 | 1 |
| LPC | 1.04 (1.00, 1.08) | | 0.03 | 0.10 | 1 | 0.95 (0.92, 0.99) | 0.01 | 0.05 | 0.59 | 1.00 (0.96, 1.04) | 0.90 | 0.90 | 1 |
| PE | 0.97 (0.93, 1.00) | | 0.08 | 0.18 | 1 | 1.03 (0.99, 1.07) | 0.10 | 0.21 | 1 | 0.99 (0.96, 1.03) | 0.76 | 0.87 | 1 |
| LPE | 1.01 (0.97, 1.05) | | 0.69 | 0.87 | 1 | 0.97 (0.93, 1.00) | 0.06 | 0.16 | 1 | 0.98 (0.94, 1.01) | 0.21 | 0.39 | 1 |
| PI | 0.99 (0.96, 1.03) | | 0.76 | 0.87 | 1 | 1.00 (0.96, 1.03) | 0.88 | 0.90 | 1 | 0.99 (0.96, 1.03) | 0.75 | 0.87 | 1 |
| CER | 0.94 (0.91, 0.98) | | 0.002 | 0.01 | 0.08 | 1.05 (1.01, 1.09) | 0.02 | 0.05 | 0.64 | 0.98 (0.94, 1.02) | 0.28 | 0.51 | 1 |
| DCER | **0.92 (0.89, 0.96)** | | **2.6E-05** | **0.0005** | **0.001** | **1.07 (1.03, 1.11)** | **0.0002** | **0.001** | **0.01** | 0.98 (0.94, 1.02) | 0.30 | 0.52 | 1 |
| HCER | 1.04 (1.00, 1.08) | | 0.04 | 0.12 | 1 | 0.96 (0.93, 1.00) | 0.03 | 0.09 | 1 | 1.00 (0.97, 1.04) | 0.85 | 0.90 | 1 |
| LCER | **1.06 (1.03, 1.10)** | | **0.001** | **0.01** | **0.05** | 0.95 (0.91, 0.98) | 0.003 | 0.01 | 0.11 | 1.01 (0.98, 1.05) | 0.44 | 0.63 | 1 |
| SM | 0.97 (0.93, 1.00) | | 0.08 | 0.19 | 1 | 1.03 (0.99, 1.07) | 0.15 | 0.29 | 1 | 0.99 (0.96, 1.03) | 0.70 | 0.87 | 1 |
| CE | 0.95 (0.92, 0.99) | | 0.01 | 0.05 | 0.61 | 1.03 (1.00, 1.07) | 0.07 | 0.18 | 1 | 0.98 (0.95, 1.02) | 0.33 | 0.53 | 1 |
| DAG | **0.92 (0.89, 0.96)** | | **5.9E-05** | **0.0006** | **0.002** | **1.08 (1.04, 1.12)** | **0.0001** | **0.0009** | **0.005** | 0.99 (0.95, 1.03) | 0.52 | 0.70 | 1 |
| TAG | **0.92 (0.89, 0.96)** | | **4.3E-05** | **0.0006** | **0.002** | **1.09 (1.05, 1.13)** | **2.1E-05** | **0.0005** | **0.0009** | 0.99 (0.96, 1.03) | 0.78 | 0.87 | 1 |
| MAG | 1.01 (0.98, 1.05) | | 0.48 | 0.67 | 1 | 1.00 (0.97, 1.04) | 0.89 | 0.90 | 1 | 1.02 (0.98, 1.05) | 0.35 | 0.55 | 1 |
| a. Models were adjusted for age (continuous), age at menarche (continuous), body fat % (continuous), race (non-Hispanic white, non-Hispanic black, other), family history of breast cancer (yes, no), oral contraceptive use (never, less than 1 year, 1-4 years, 5-9 years, more than 10 years), alcohol consumption (never, less than 1 drink per week, 1-2 drinks per week, 3-5 drinks per week, and 6+ drinks per week), parity/age at first birth (nulliparous, 1-2 children & < 25 years, 1-2 children & 25-29 years, 1-2 children & , ≥30 years, ≥3 children & < 25 years, ≥3 children & ≥25 years) and body shape at age 10 (1&2, 3&4, 5, 6-9).  b. Mammographic breast density measures were log_10_ transformed and coefficients were back-transformed (10^β) and bolded if the Bonferroni p-trend is <0.05.  Abbreviations: phosphatidylcholine (PC), lysophosphatidylcholine (LPC), phosphatidylethanolamine (PE), lysophosphatidylethanolamine (LPE), phosphatidylinositol (PI), ceramide (CER), dihydroceramide (DCER), hexosylceramide (HCER), lactosylceramide (LCER), sphingomyelin (SM), cholesteryl ester (CE), diacylglycerol (DAG), triacylglycerol (TAG), monoacylglycerol (MAG), odds ratio (OR), confidence interval (CI), false discovery rate (FDR), centimeters (cm). | | | | | | | | | | | | | |

**Supplementary Table 3: Covariate-adjusted Least Square Means of Non-dense Volume (NDV) by Quartiles of Lipid Species that were Significantly Associated with NDV at a Bonferroni P-value<0.05**

|  | **Q1**  **LSM (95% CI)** | | **Q2**  **LSM (95% CI)** | **Q3**  **LSM (95% CI)** | **Q4**  **LSM (95% CI)** | **p-trend** | **FDR**  **p-value** | **Bonferroni**  **p-value** | | |
| --- | --- | --- | --- | --- | --- | --- | --- | --- | --- | --- |
| *Back-transformed* ^a,b,c^ | | |  |  |  |  |  |  | |  |
| LPC(18:1) | | 917.6 (827.3-1017.7) | 881.3 (797.5-973.9) | 778.4 (703.5-861.3) | 740.9 (668.4-821.3) | 3.5E-05 | 0.0003 | 0.09 | |  |
| PC(18:1/18:1) | | 910.9 (822.4-1008.9) | 827.2 (750.6-911.5) | 836.2 (752.8-928.9) | 714.3 (643.2-793.2) | 2.1E-05 | 0.0002 | 0.05 | |  |
| PI(18:1/18:1) | | 908.8 (820.7-1006.4) | 869.6 (786.7-961.3) | 793.8 (718.7-876.8) | 740.6 (668.3-820.7) | 5.3E-05 | 0.0004 | 0.14 | |  |
| **LCER(14:0)** | | **905.2 (820.1-999.1)** | **837.7 (758.1-925.6)** | **799.0 (721.0-885.5)** | **708.6 (636.4-788.9)** | **1.2E-05** | **0.0001** | **0.03** | |  |
| **DAG(16:0/18:2)** | | **693.8 (626.8-767.9)** | **792.4 (718.1-874.4)** | **921.8 (834.7-1017.9)** | **918.8 (829.2-1018.0)** | **6.0E-06** | **8.4E-05** | **0.02** | |  |
| **DAG(16:0/18:3)** | | **721.0 (653.0-796.1)** | **773.6 (699.1-856.1)** | **884.8 (797.9-981.1)** | **946.6 (856.9-1045.6)** | **1.0E-06** | **3.3E-05** | **0.003** | |  |
| DAG(16:0/20:3) | | 755.5 (682.2-836.7) | 764.0 (691.7-843.7) | 881.1 (797.4-973.6) | 922.1 (831.2-1022.8) | 7.6E-05 | 0.0005 | 0.19 | |  |
| DAG(16:0/20:4) | | 736.4 (663.7-817.1) | 772.8 (699.8-853.3) | 872.6 (788.8-965.3) | 926.3 (836.5-1025.8) | 3.8E-05 | 0.0003 | 0.10 | |  |
| **DAG(16:1/18:1)** | | **736.6 (666.7-813.8)** | **813.1 (736.7-897.4)** | **834.1 (752.0-925.1)** | **971.1 (873.6-1079.5)** | **2.6E-06** | **5.2E-05** | **0.007** | |  |
| **DAG(16:1/18:2)** | | **715.5 (646.3-792.1)** | **829.0 (752.4-913.5)** | **862.8 (780.0-954.4)** | **944.2 (847.4-1052.1)** | **1.6E-05** | 0.0002 | **0.04** | |  |
| **DAG(16:1/18:3)** | | **733.4 (663.9-810.2)** | **781.6 (707.7-863.1)** | **875.6 (790.5-970.0)** | **960.6 (866.1-1065.3)** | **1.2E-06** | **3.6E-05** | **0.003** | |  |
| DAG(16:1/20:2) | | 749.3 (677.4-828.9) | 800.3 (724.1-884.5) | 838.6 (757.8-928.0) | 943.8 (849.6-1048.6) | 3.0E-05 | 0.0002 | 0.08 | |  |
| **DAG(16:1/20:4)** | | **757.0 (684.7-837.1)** | **797.7 (720.6-882.9)** | **828.9 (750.0-916.0)** | **960.7 (864.6-1067.5)** | **9.8E-06** | **0.0001** | **0.03** | |  |
| DAG(18:0/18:3) | | 737.5 (666.5-816.1) | 800.7 (725.4-883.8) | 853.4 (768.6-947.6) | 925.6 (836.8-1024.0) | 5.2E-05 | 0.0004 | 0.14 | |  |
| **DAG(18:1/20:4)** | | **774.0 (699.8-856.1)** | **778.1 (703.3-860.7)** | **815.5 (737.5-901.6)** | **959.2 (864.8-1063.9)** | **1.0E-05** | **0.0001** | **0.03** | |  |
| DAG(18:2/20:3) | | 746.4 (674.5-826.0) | 823.7 (746.0-909.5) | 854.7 (772.6-945.7) | 916.3 (824.2-1018.8) | 0.0003 | 0.002 | 0.83 | |  |
| **DAG(18:2/20:4)** | | **731.2 (660.6-809.3)** | **800.4 (723.6-885.4)** | **834.2 (753.7-923.3)** | **958.4 (865.6-1061.2)** | **9.8E-07** | **3.2E-05** | **0.003** | |  |
| DAG(18:2/22:5) | | 724.6 (655.2-801.3) | 839.1 (760.8-925.4) | 858.2 (775.9-949.3) | 920.3 (828.1-1022.8) | 7.5E-05 | 0.0005 | 0.19 | |  |
| **TAG49:3-FA15:0** | | **742.9 (674.3-818.5)** | **814.8 (738.3-899.3)** | **816.2 (736.4-904.7)** | **1003.2 (903.6-1113.9)** | **2.8E-08** | **5.2E-06** | **7.2E-05** | |  |
| **TAG49:3-FA16:0** | | **765.3 (693.1-845.0)** | **770.0 (697.0-850.6)** | **846.0 (764.1-936.7)** | **963.0 (868.1-1068.2)** | **2.3E-06** | **4.9E-05** | **0.006** | |  |
| **TAG49:3-FA16:1** | | **742.9 (673.9-819.0)** | **837.0 (759.3-922.7)** | **838.3 (755.8-929.8)** | **976.8 (876.3-1088.8)** | **4.3E-06** | **6.8E-05** | **0.01** | |  |
| **TAG50:2-FA16:0** | | **705.3 (637.0-780.9)** | **789.8 (716.7-870.4)** | **880.0 (794.5-974.6)** | **951.8 (859.7-1053.9)** | **4.3E-07** | **2.1E-05** | **0.001** | |  |
| **TAG50:2-FA18:2** | | **700.2 (633.2-774.3)** | **775.1 (701.8-856.1)** | **906.6 (820.9-1001.3)** | **932.7 (843.5-1031.2)** | **6.6E-07** | **2.7E-05** | **0.002** | |  |
| **TAG50:3-FA16:0** | | **699.1 (632.0-773.3)** | **788.7 (715.2-869.7)** | **886.2 (802.0-979.3)** | **971.8 (876.3-1077.7)** | **5.1E-08** | **6.5E-06** | **0.0001** | |  |
| **TAG50:3-FA16:1** | | **719.6 (651.1-795.2)** | **794.2 (720.5-875.5)** | **877.8 (794.0-970.5)** | **982.7 (883.2-1093.3)** | **1.6E-07** | **1.2E-05** | **0.0004** | |  |
| **TAG50:3-FA18:2** | | **708.9 (641.9-783.0)** | **808.1 (731.8-892.3)** | **880.9 (797.5-973.0)** | **969.1 (872.3-1076.7)** | **1.5E-07** | **1.2E-05** | **0.0004** | |  |
| **TAG50:3-FA18:3** | | **734.4 (664.2-812.0)** | **757.5 (684.8-837.9)** | **890.7 (805.8-984.5)** | **927.2 (838.0-1025.8)** | **1.1E-05** | **0.0001** | **0.03** | |  |
| **TAG50:3-FA20:3** | | **765.0 (693.0-844.4)** | **773.8 (699.8-855.6)** | **824.5 (744.1-913.7)** | **956.1 (863.7-1058.3)** | **3.1E-06** | **5.6E-05** | **0.008** | |  |
| **TAG50:4-FA16:0** | | **701.0 (634.9-774.0)** | **789.9 (714.7-872.9)** | **876.9 (791.9-970.9)** | **973.7 (880.5-1076.8)** | **3.1E-08** | **5.4E-06** | **8.1E-05** | |  |
| **TAG50:4-FA18:2** | | **732.2 (664.0-807.5)** | **829.6 (750.4-917.3)** | **865.5 (781.8-958.1)** | **944.6 (849.9-1049.8)** | **1.1E-05** | **0.0001** | **0.03** | |  |
| **TAG50:4-FA18:3** | | **735.1 (666.8-810.3)** | **811.4 (732.3-899.1)** | **827.1 (746.3-916.5)** | **970.7 (876.6-1075.0)** | **5.8E-07** | **2.5E-05** | **0.002** | |  |
| **TAG50:5-FA16:0** | | **701.8 (635.3-775.4)** | **811.7 (734.6-897.0)** | **868.0 (782.3-963.0)** | **947.9 (858.0-1047.1)** | **1.8E-06** | **4.4E-05** | **0.005** | |  |
| **TAG51:2-FA16:0** | | **725.7 (656.4-802.4)** | **811.0 (736.4-893.1)** | **849.6 (767.6-940.5)** | **978.1 (880.2-1086.9)** | **2.2E-07** | **1.4E-05** | **0.001** | |  |
| **TAG51:2-FA17:0** | | **725.7 (656.4-802.4)** | **817.1 (741.3-900.7)** | **822.5 (743.3-910.1)** | **977.3 (881.2-1083.8)** | **1.8E-07** | **1.3E-05** | **0.0005** | |  |
| **TAG51:3-FA16:0** | | **700.4 (634.0-773.8)** | **808.6 (732.5-892.7)** | **879.9 (795.5-973.2)** | **968.4 (873.4-1073.7)** | **9.3E-08** | **9.1E-06** | **0.0002** | |  |
| **TAG51:3-FA17:0** | | **754.3 (682.9-833.1)** | **794.1 (719.1-876.9)** | **847.4 (764.9-938.8)** | **959.5 (863.5-1066.1)** | **7.2E-06** | **9.5E-05** | **0.02** | |  |
| **TAG51:3-FA18:2** | | **727.6 (659.2-803.2)** | **843.1 (763.8-930.7)** | **851.9 (769.4-943.2)** | **955.1 (858.6-1062.4)** | **8.1E-06** | **0.0001** | **0.02** | |  |
| **TAG51:4-FA16:0** | | **704.6 (637.5-778.7)** | **802.5 (728.0-884.6)** | **857.8 (774.4-950.2)** | **976.2 (882.0-1080.5)** | **1.5E-08** | **3.7E-06** | **3.7E-05** | |  |
| **TAG51:4-FA16:1** | | **735.7 (666.8-811.7)** | **802.2 (727.0-885.2)** | **901.3 (814.1-997.7)** | **945.5 (848.1-1054.1)** | **1.7E-05** | **0.0002** | **0.04** | |  |
| **TAG52:2-FA18:2** | | **703.9 (635.9-779.1)** | **791.5 (717.7-872.9)** | **905.4 (819.3-1000.4)** | **928.9 (838.4-1029.2)** | **3.1E-06** | **5.6E-05** | **0.008** | |  |
| **TAG52:3-FA16:0** | | **694.2 (627.2-768.3)** | **810.0 (734.4-893.4)** | **898.7 (812.6-993.9)** | **928.6 (838.6-1028.3)** | **1.1E-06** | **3.3E-05** | **0.003** | |  |
| **TAG52:3-FA18:1** | | **707.4 (640.1-781.7)** | **815.2 (738.7-899.6)** | **883.6 (797.5-979.0)** | **937.1 (846.0-1038.1)** | **1.4E-06** | **3.9E-05** | **0.004** | |  |
| **TAG52:3-FA18:2** | | **701.5 (633.9-776.3)** | **808.0 (732.9-890.7)** | **901.7 (814.8-998.0)** | **923.0 (833.3-1022.3)** | **3.2E-06** | **5.6E-05** | **0.008** | |  |
| **TAG52:3-FA18:3** | | **734.9 (664.7-812.6)** | **757.4 (683.9-838.9)** | **884.7 (800.2-978.2)** | **929.0 (840.0-1027.5)** | **6.9E-06** | **9.4E-05** | **0.02** | |  |
| **TAG52:3-FA20:0** | | **713.4 (644.7-789.4)** | **785.3 (711.6-866.6)** | **901.0 (814.6-996.5)** | **933.4 (842.6-1034.0)** | **2.1E-06** | **4.7E-05** | **0.005** | |  |
| **TAG52:3-FA20:2** | | **699.7 (633.8-772.5)** | **857.2 (777.1-945.4)** | **837.3 (757.1-926.0)** | **970.6 (874.5-1077.2)** | **8.0E-07** | **3.0E-05** | **0.002** | |  |
| **TAG52:3-FA20:3** | | **748.7 (676.5-828.5)** | **757.8 (686.8-836.3)** | **863.9 (780.4-956.2)** | **957.0 (863.7-1060.4)** | **1.6E-06** | **4.2E-05** | **0.004** | |  |
| **TAG52:3-FA22:1** | | **719.1 (649.8-795.8)** | **782.9 (708.5-865.2)** | **891.9 (807.1-985.6)** | **929.4 (838.5-1030.2)** | **5.8E-06** | **8.3E-05** | **0.02** | |  |
| **TAG52:4-FA14:0** | | **725.7 (659.0-799.1)** | **829.6 (750.8-916.7)** | **838.9 (757.1-929.6)** | **986.7 (889.9-1093.9)** | **5.5E-08** | **6.7E-06** | **0.0001** | |  |
| **TAG52:4-FA16:0** | | **718.6 (649.7-794.8)** | **775.2 (701.4-856.8)** | **889.3 (805.0-982.4)** | **940.7 (849.9-1041.1)** | **3.0E-07** | **1.7E-05** | **0.001** | |  |
| **TAG52:4-FA18:2** | | **723.1 (654.0-799.5)** | **790.3 (714.4-874.3)** | **881.5 (797.9-974.0)** | **931.4 (840.9-1031.6)** | **2.4E-06** | **5.0E-05** | **0.006** | |  |
| **TAG52:4-FA18:3** | | **735.3 (665.1-812.9)** | **752.1 (679.1-832.8)** | **887.1 (801.2-982.3)** | **930.9 (842.2-1028.8)** | **3.5E-06** | **6.1E-05** | **0.009** | |  |
| **TAG52:4-FA20:0** | | **709.1 (641.3-784.2)** | **777.0 (703.2-858.5)** | **895.4 (810.3-989.5)** | **940.3 (850.5-1039.5)** | **1.2E-07** | **1.1E-05** | **0.0003** | |  |
| **TAG52:4-FA20:2** | | **717.8 (650.4-792.2)** | **835.1 (755.2-923.5)** | **872.7 (790.1-964.1)** | **943.8 (848.3-1050.1)** | **6.0E-06** | **8.4E-05** | **0.02** | |  |
| **TAG52:4-FA20:3** | | **726.2 (657.8-801.9)** | **797.4 (721.9-880.8)** | **849.9 (767.9-940.5)** | **980.8 (884.4-1087.6)** | **1.1E-07** | **1.0E-05** | **0.0003** | |  |
| **TAG52:4-FA20:4** | | **723.2 (652.8-801.3)** | **759.6 (685.9-841.3)** | **857.3 (776.9-946.1)** | **948.3 (856.9-1049.5)** | **7.0E-07** | **2.7E-05** | **0.002** | |  |
| **TAG52:4-FA22:1** | | **712.2 (644.5-786.9)** | **803.9 (727.1-888.8)** | **885.1 (799.8-979.4)** | **935.4 (844.6-1036.0)** | **1.8E-06** | **4.5E-05** | **0.005** | |  |
| **TAG52:4-FA22:4** | | **702.3 (636.3-775.2)** | **814.1 (737.0-899.2)** | **860.5 (778.4-951.4)** | **972.1 (878.7-1075.5)** | **4.7E-08** | **6.5E-06** | **0.0001** | |  |
| **TAG52:5-FA14:0** | | **738.8 (669.5-815.2)** | **790.1 (716.3-871.5)** | **820.1 (739.7-909.2)** | **995.6 (899.5-1101.9)** | **6.8E-09** | **3.4E-06** | **1.8E-05** | |  |
| **TAG52:5-FA16:0** | | **729.8 (659.9-807.2)** | **775.9 (701.2-858.7)** | **874.8 (790.2-968.5)** | **930.3 (841.5-1028.5)** | **4.4E-06** | **7.0E-05** | **0.01** | |  |
| **TAG52:5-FA18:3** | | **716.8 (647.7-793.2)** | **793.0 (715.3-879.1)** | **872.9 (790.3-964.1)** | **920.0 (831.8-1017.6)** | **8.1E-06** | **0.0001** | **0.02** | |  |
| **TAG52:5-FA20:3** | | **743.5 (675.2-818.8)** | **806.6 (729.4-891.8)** | **852.7 (769.2-945.3)** | **967.7 (872.3-1073.5)** | **8.2E-07** | **3.0E-05** | **0.002** | |  |
| **TAG52:5-FA20:4** | | **739.1 (668.9-816.5)** | **756.1 (683.3-836.5)** | **861.9 (778.9-953.7)** | **967.1 (873.3-1070.9)** | **2.5E-07** | **1.5E-05** | **0.0006** | |  |
| **TAG52:5-FA22:5** | | **714.0 (646.5-788.6)** | **785.3 (710.3-868.1)** | **863.9 (781.6-954.8)** | **979.0 (884.7-1083.3)** | **8.3E-09** | **3.4E-06** | **2.1E-05** | |  |
| **TAG52:6-FA14:0** | | **738.2 (667.8-816.1)** | **787.7 (713.6-869.5)** | **846.8 (763.6-939.1)** | **953.3 (861.6-1054.7)** | **1.4E-06** | **3.9E-05** | **0.004** | |  |
| TAG52:6-FA16:0 | | 738.2 (667.3-816.7) | 780.1 (704.8-863.4) | 864.4 (781.1-956.7) | 922.1 (834.1-1019.5) | 3.0E-05 | 0.0002 | 0.08 | |  |
| **TAG52:6-FA20:4** | | **741.2 (671.2-818.5)** | **784.4 (709.2-867.6)** | **864.4 (780.2-957.7)** | **950.6 (857.6-1053.6)** | **3.6E-06** | **6.1E-05** | **0.009** | |  |
| TAG53:0-FA16:0 | | 720.4 (650.1-798.3) | 770.7 (697.5-851.6) | 900.7 (812.7-998.2) | 920.0 (833.1-1016.0) | 2.5E-05 | 0.0002 | 0.06 | |  |
| **TAG53:3-FA16:0** | | **731.8 (662.4-808.4)** | **751.1 (680.2-829.5)** | **918.7 (831.6-1015.0)** | **932.2 (841.2-1033.1)** | **2.1E-06** | **4.7E-05** | **0.005** | |  |
| **TAG53:4-FA16:0** | | **696.5 (629.4-770.6)** | **811.5 (735.9-894.9)** | **865.3 (782.8-956.4)** | **957.3 (864.9-1059.6)** | **7.2E-08** | **8.1E-06** | **0.0002** | |  |
| **TAG53:4-FA20:4** | | **740.1 (668.9-818.9)** | **797.5 (723.6-879.0)** | **816.7 (736.7-905.4)** | **968.5 (874.3-1072.8)** | **4.5E-07** | **2.2E-05** | **0.001** | |  |
| **TAG53:5-FA20:4** | | **731.4 (662.6-807.2)** | **819.0 (742.2-903.6)** | **854.3 (770.2-947.6)** | **956.4 (861.6-1061.6)** | **3.6E-06** | **6.1E-05** | **0.009** | |  |
| **TAG53:6-FA20:4** | | **750.0 (678.0-829.6)** | **785.0 (713.1-864.2)** | **863.4 (777.2-959.2)** | **958.0 (863.6-1062.8)** | **2.6E-06** | **5.2E-05** | **0.007** | |  |
| TAG54:3-FA16:0 | | 727.5 (657.7-804.6) | 836.0 (759.1-920.7) | 841.0 (758.0-933.1) | 934.5 (841.2-1038.3) | 4.4E-05 | 0.0003 | 0.11 | |  |
| **TAG54:3-FA20:3** | | **730.2 (660.1-807.7)** | **785.2 (712.2-865.7)** | **846.0 (763.8-937.2)** | **969.5 (875.3-1073.9)** | **1.8E-07** | **1.3E-05** | **0.0005** | |  |
| **TAG54:4-FA16:0** | | **713.6 (646.2-788.2)** | **812.5 (736.9-896.0)** | **882.4 (796.3-977.8)** | **956.0 (861.4-1060.9)** | **1.1E-06** | **3.3E-05** | **0.003** | |  |
| **TAG54:4-FA20:2** | | **710.9 (643.5-785.4)** | **849.3 (769.4-937.5)** | **835.6 (754.7-925.3)** | **947.5 (854.5-1050.6)** | **3.5E-06** | **6.1E-05** | **0.009** | |  |
| **TAG54:4-FA20:3** | | **731.2 (661.5-808.2)** | **782.1 (709.3-862.3)** | **871.7 (786.2-966.4)** | **965.6 (871.3-1070.1)** | **3.4E-07** | **1.9E-05** | **0.001** | |  |
| **TAG54:4-FA20:4** | | **716.6 (646.3-794.4)** | **764.4 (693.5-842.6)** | **890.7 (804.8-985.6)** | **949.8 (857.8-1051.6)** | **5.7E-07** | **2.5E-05** | **0.001** | |  |
| **TAG54:4-FA22:4** | | **714.8 (645.3-791.8)** | **766.0 (694.8-844.5)** | **885.6 (802.0-977.9)** | **958.3 (864.6-1062.1)** | **4.3E-07** | **2.1E-05** | **0.001** | |  |
| **TAG54:5-FA16:0** | | **701.9 (634.8-776.1)** | **782.3 (709.1-863.0)** | **881.0 (795.5-975.7)** | **989.5 (893.8-1095.5)** | **2.9E-09** | **3.4E-06** | **7.4E-06** | |  |
| TAG54:5-FA16:1 | | 738.5 (670.4-813.5) | 786.3 (709.4-871.6) | 912.1 (824.5-1009.1) | 924.2 (831.2-1027.6) | 7.8E-05 | 0.0005 | 0.2 | |  |
| **TAG54:5-FA20:3** | | **704.0 (637.8-777.2)** | **803.6 (727.5-887.7)** | **884.3 (799.5-978.1)** | **974.1 (878.9-1079.8)** | **2.1E-08** | **4.6E-06** | **5.5E-05** | |  |
| **TAG54:5-FA20:4** | | **703.3 (634.6-779.3)** | **761.1 (690.6-838.7)** | **916.3 (827.7-1014.5)** | **953.8 (862.8-1054.4)** | **1.4E-07** | **1.1E-05** | **0.0004** | |  |
| **TAG54:5-FA22:4** | | **713.8 (646.1-788.6)** | **837.7 (758.6-925.2)** | **851.9 (770.2-942.3)** | **957.3 (861.6-1063.5)** | **8.1E-06** | **0.0001** | **0.02** | |  |
| **TAG54:5-FA22:5** | | **682.5 (616.5-755.5)** | **781.6 (709.1-861.5)** | **921.5 (834.6-1017.4)** | **933.3 (843.2-1033.0)** | **1.7E-06** | **4.2E-05** | **0.004** | |  |
| **TAG54:6-FA16:0** | | **680.4 (614.8-753.0)** | **800.6 (725.3-883.8)** | **886.6 (801.2-981.1)** | **968.8 (876.4-1070.9)** | **1.0E-08** | **3.4E-06** | **2.7E-05** | |  |
| **TAG54:6-FA16:1** | | **715.8 (647.9-790.9)** | **810.1 (733.2-895.0)** | **884.7 (799.1-979.5)** | **945.8 (852.3-1049.5)** | **4.7E-06** | **7.3E-05** | **0.01** | |  |
| **TAG54:6-FA20:3** | | **716.9 (650.1-790.4)** | **843.9 (763.8-932.4)** | **851.5 (768.2-943.9)** | **958.0 (863.7-1062.7)** | **1.2E-06** | **3.5E-05** | **0.003** | |  |
| **TAG54:6-FA20:4** | | **690.9 (624.4-764.5)** | **784.7 (710.4-866.7)** | **886.5 (799.5-983.1)** | **976.6 (883.6-1079.5)** | **6.1E-09** | **3.4E-06** | **1.6E-05** | |  |
| **TAG54:6-FA22:5** | | **693.2 (626.8-766.7)** | **843.6 (764.3-931.2)** | **846.6 (766.9-934.5)** | **961.8 (867.3-1066.7)** | **6.8E-07** | **2.7E-05** | **0.002** | |  |
| **TAG54:7-FA16:1** | | **701.4 (633.7-776.3)** | **815.2 (737.9-900.6)** | **856.6 (774.4-947.5)** | **970.7 (876.1-1075.6)** | **8.6E-08** | **8.9E-06** | **0.0002** | |  |
| **TAG54:7-FA20:4** | | **710.4 (642.7-785.3)** | **781.2 (707.3-862.8)** | **882.8 (796.5-978.4)** | **993.0 (896.0-1100.5)** | **4.0E-09** | **3.4E-06** | **1.0E-05** | |  |
| **TAG54:7-FA22:5** | | **719.9 (652.6-794.0)** | **847.6 (766.2-937.6)** | **865.1 (782.9-956.0)** | **947.9 (852.8-1053.5)** | **7.0E-06** | **9.4E-05** | **0.02** | |  |
| TAG54:8-FA20:4 | | 727.8 (658.2-804.8) | 827.5 (748.0-915.4) | 830.2 (748.3-921.2) | 938.0 (847.2-1038.4) | 3.0E-05 | 0.0002 | 0.08 | |  |
| **TAG55:1-FA16:0** | | **718.0 (649.0-794.4)** | **801.9 (726.5-885.2)** | **880.4 (793.6-976.8)** | **919.6 (832.5-1015.7)** | **1.6E-05** | **0.0002** | **0.04** | |  |
| **TAG55:5-FA20:4** | | **717.7 (649.4-793.2)** | **836.6 (757.2-924.3)** | **843.4 (762.7-932.7)** | **949.3 (856.4-1052.3)** | **2.9E-06** | **5.4E-05** | **0.007** | |  |
| **TAG55:6-FA20:4** | | **720.4 (652.8-795.0)** | **827.6 (749.3-914.0)** | **840.1 (757.0-932.3)** | **973.0 (877.8-1078.5)** | **2.0E-07** | **1.3E-05** | **0.0005** | |  |
| **TAG55:7-FA20:4** | | **748.4 (676.1-828.3)** | **779.6 (707.7-858.8)** | **808.4 (732.8-891.8)** | **1006.7 (908.7-1115.4)** | **8.8E-09** | **3.4E-06** | **2.3E-05** | |  |
| TAG56:3-FA16:0 | | 720.8 (652.6-796.1) | 861.5 (778.8-953.1) | 837.8 (757.5-926.5) | 923.9 (833.8-1023.8) | 0.0002 | 0.001 | 0.53 | |  |
| **TAG56:4-FA16:0** | | **686.2 (620.7-758.6)** | **849.7 (770.2-937.3)** | **857.3 (775.1-948.2)** | **936.0 (845.6-1036.0)** | **3.3E-06** | **5.8E-05** | **0.009** | |  |
| **TAG56:4-FA22:4** | | **719.3 (648.6-797.6)** | **775.1 (703.6-854.0)** | **894.6 (808.7-989.5)** | **944.0 (851.6-1046.4)** | **2.3E-06** | **4.9E-05** | **0.006** | |  |
| **TAG56:5-FA16:0** | | **700.3 (633.1-774.7)** | **818.7 (743.3-901.7)** | **864.9 (780.2-958.7)** | **953.1 (860.5-1055.7)** | **4.1E-07** | **2.1E-05** | **0.001** | |  |
| **TAG56:5-FA18:0** | | **678.6 (613.9-750.2)** | **865.5 (785.8-953.3)** | **849.0 (767.0-939.8)** | **928.3 (839.2-1027.0)** | **1.5E-06** | **4.0E-05** | **0.004** | |  |
| **TAG56:5-FA22:4** | | **691.9 (625.0-766.1)** | **806.4 (732.4-887.9)** | **886.4 (799.7-982.5)** | **945.8 (854.9-1046.3)** | **5.5E-07** | **2.4E-05** | **0.001** | |  |
| **TAG56:6-FA16:0** | | **712.5 (643.6-788.8)** | **805.6 (730.1-888.8)** | **848.6 (766.1-940.0)** | **941.0 (851.2-1040.3)** | **1.3E-06** | **3.6E-05** | **0.003** | |  |
| **TAG56:6-FA18:0** | | **704.3 (637.8-777.8)** | **810.0 (734.1-893.9)** | **894.8 (807.3-991.8)** | **944.7 (853.9-1045.1)** | **3.4E-07** | **1.9E-05** | **0.001** | |  |
| **TAG56:6-FA18:2** | | **710.1 (642.7-784.6)** | **813.4 (736.7-898.2)** | **884.4 (798.6-979.5)** | **931.8 (842.2-1031.0)** | **1.4E-06** | **3.8E-05** | **0.004** | |  |
| **TAG56:6-FA22:4** | | **687.7 (622.4-759.9)** | **795.6 (720.9-878.0)** | **914.3 (826.2-1011.8)** | **955.1 (863.1-1057.0)** | **4.9E-08** | **6.5E-06** | **0.0001** | |  |
| **TAG56:7-FA16:0** | | **708.9 (641.2-783.7)** | **816.5 (739.1-902.0)** | **881.2 (796.7-974.7)** | **921.2 (832.4-1019.5)** | **1.3E-05** | **0.0001** | **0.03** | |  |
| **TAG56:7-FA22:4** | | **720.4 (652.1-795.8)** | **779.5 (705.4-861.4)** | **918.0 (828.7-1016.9)** | **932.3 (841.4-1033.0)** | **4.4E-06** | **7.0E-05** | **0.01** | |  |
| **TAG56:7-FA22:5** | | **699.6 (633.1-773.1)** | **808.0 (731.8-892.1)** | **900.6 (813.5-997.0)** | **931.4 (841.9-1030.5)** | **1.3E-06** | **3.6E-05** | **0.003** | |  |
| **TAG56:8-FA16:0** | | **714.3 (645.5-790.4)** | **811.0 (734.7-895.2)** | **864.0 (779.4-957.8)** | **926.5 (838.4-1023.9)** | **8.1E-06** | **0.0001** | **0.02** | |  |
| **TAG56:8-FA22:5** | | **701.3 (635.2-774.3)** | **852.8 (771.5-942.7)** | **855.8 (773.0-947.4)** | **944.2 (853.0-1045.1)** | **1.7E-06** | **4.2E-05** | **0.004** | |  |
| **TAG58:7-FA20:4** | | **730.8 (661.0-807.9)** | **827.9 (749.8-914.1)** | **844.9 (762.6-936.0)** | **943.6 (850.3-1047.2)** | **1.3E-05** | **0.0001** | **0.03** | |  |
| TAG58:8-FA20:4 | | 761.3 (687.9-842.6) | 756.2 (682.3-838.1) | 858.4 (776.6-948.8) | 932.9 (840.6-1035.3) | 4.7E-05 | 0.0003 | 0.12 | |  |
| **TAG58:9-FA20:4** | | **739.7 (667.1-820.1)** | **784.9 (709.4-868.4)** | **820.1 (742.9-905.4)** | **964.5 (870.0-1069.4)** | **1.0E-06** | **3.3E-05** | **0.003** | |  |
| a. Models were adjusted for age (continuous), age at menarche (continuous), body fat % (continuous), race (non-Hispanic white, non-Hispanic black, other), family history of breast cancer (yes, no), oral contraceptive use (never, less than 1 year, 1-4 years, 5-9 years, more than 10 years), alcohol consumption (never, less than 1 drink per week, 1-2 drinks per week, 3-5 drinks per week, and 6+ drinks per week), parity/age at first birth (nulliparous, 1-2 children & < 25 years, 1-2 children & 25-29 years, 1-2 children & , ≥30 years, ≥3 children & < 25 years, ≥3 children & ≥25 years) and body shape at age 10 (1&2, 3&4, 5, 6-9).  b. Non-dense volume was log_10_ transformed and coefficients were back-transformed (10^β) and bolded if the Bonferroni p-trend is <0.05.  c. Lipid species presented were associated with non-dense volume in multivariable linear regression analyses, after Bonferroni correction  Abbreviations: phosphatidylcholine (PC), lysophosphatidylcholine (LPC), phosphatidylinositol (PI), lactosylceramide (LCER), diacylglycerol (DAG), triacylglycerol (TAG), quartiles 1-4 (Q1-4), least square mean (LSM), confidence interval (CI), false discovery rate (FDR). | | | | | | | | |  |  |

**Supplementary Table 4: Covariate-adjusted Least Square Mean of Dense Volume and Non-dense Volume by Quartile of Lipid Sub-pathways**

|  | **Q1**  **LSM (95% CI)** | **Q2**  **LSM (95% CI)** | **Q3**  **LSM (95% CI)** | **Q4**  **LSM (95% CI)** | | **p-trend** | | **FDR**  **p-value** | **Bonferroni**  **p-value** | | |
| --- | --- | --- | --- | --- | --- | --- | --- | --- | --- | --- | --- |
| *Non-dense Volume (cm^3^)* ^a,b,c^ | |  |  |  | |  | |  |  | | |
| PC | 819.1 (739.9-906.8) | 785.8 (710.0-869.7) | 858.3 (775.1-950.3) | 842.6 (758.3-936.4) | | 0.36 | | 0.63 | 1 | | |
| LPC | 882.1 (795.9-977.7) | 819.2 (739.2-907.8) | 810.7 (733.4-896.2) | 786.0 (708.6-871.7) | | 0.04 | | 0.14 | 1 | | |
| PE | 761.3 (689.6-840.4) | 873.1 (790.1-964.8) | 868.7 (782.6-964.3) | 818.7 (739.7-906.1) | | 0.29 | | 0.55 | 1 | | |
| LPE | 835.6 (755.8-923.8) | 868.6 (783.5-963.0) | 819.9 (741.0-907.2) | 781.1 (704.9-865.6) | | 0.10 | | 0.25 | 1 | | |
| PI | 803.7 (727.5-887.9) | 856.4 (772.6-949.3) | 839.8 (759.5-928.6) | 803.2 (723.9-891.3) | | 0.77 | | 0.81 | 1 | | |
| CER | 786.0 (711.3-868.6) | 784.2 (708.7-867.8) | 883.9 (798.8-978.1) | 860.1 (775.3-954.2) | | 0.03 | | 0.11 | 1 | | |
| **DCER** | **754.0 (682.0-833.7)** | **823.8 (745.2-910.7)** | **840.3 (759.9-929.2)** | **909.4 (818.8-1010.1)** | | **0.0007** | | **0.003** | **0.03** | | |
| HCER | 863.2 (781.1-954.0) | 805.0 (728.0-890.2) | 832.5 (750.2-923.8) | 797.0 (719.3-883.1) | | 0.19 | | 0.42 | 1 | | |
| **LCER** | **903.9 (818.4-998.4)** | **840.6 (760.9-928.6)** | **785.7 (709.9-869.6)** | **747.7 (673.4-830.2)** | | **0.0003** | | **0.002** | **0.01** | | |
| SM | 799.0 (722.9-883.1) | 826.1 (747.0-913.6) | 831.3 (751.0-920.0) | 853.9 (769.0-948.1) | | 0.21 | | 0.44 | 1 | | |
| CE | 812.4 (734.7-898.4) | 784.1 (710.2-865.8) | 826.6 (746.3-915.4) | 900.5 (811.3-999.5) | | 0.02 | | 0.09 | 0.98 | | |
| **DAG** | **764.1 (691.1-844.8)** | **796.2 (719.4-881.1)** | **843.9 (762.0-934.6)** | **917.9 (827.6-1018.2)** | | **0.0005** | | **0.003** | **0.02** | | |
| **TAG** | **732.3 (661.9-810.2)** | **786.8 (711.1-870.6)** | **870.7 (788.4-961.5)** | **932.7 (841.0-1034.3)** | | **1.3E-05** | | **0.0003** | **0.0006** | | |
| MAG | 802.7 (725.8-887.7) | 832.4 (752.9-920.3) | 837.4 (756.0-927.5) | 829.9 (748.5-920.1) | | 0.77 | | 0.81 | 1 | | |
| *Dense Volume (cm^3^)* ^a,b,c^ | |  |  |  |  | |  | |  | | |
| PC | 72.7 (65.6-80.5) | 68.2 (61.6-75.5) | 69.6 (62.9-77.1) | 71.2 (64.0-79.1) | 0.90 | | 0.92 | | | 1 |  |
| LPC | 69.1 (62.3-76.6) | 72.0 (65.0-79.9) | 71.2 (64.4-78.8) | 69.4 (62.6-77.1) | 0.96 | | 0.96 | | | 1 |  |
| PE | 68.5 (62.1-75.7) | 72.6 (65.7-80.3) | 74.6 (67.2-82.9) | 67.2 (60.7-74.5) | 0.63 | | 0.71 | | | 1 |  |
| LPE | 69.7 (63.0-77.1) | 73.0 (65.9-81.0) | 71.1 (64.2-78.7) | 68.3 (61.6-75.8) | 0.52 | | 0.66 | | | 1 |  |
| PI | 69.5 (62.9-76.8) | 74.2 (67.0-82.3) | 71.2 (64.4-78.7) | 66.9 (60.3-74.2) | 0.27 | | 0.53 | | | 1 |  |
| CER | 74.0 (66.9-81.9) | 68.0 (61.4-75.2) | 69.3 (62.6-76.7) | 69.8 (62.9-77.5) | 0.41 | | 0.63 | | | 1 |  |
| DCER | 72.9 (65.8-80.7) | 70.2 (63.4-77.7) | 68.2 (61.6-75.5) | 70.4 (63.3-78.3) | 0.57 | | 0.70 | | | 1 |  |
| HCER | 68.4 (61.8-75.6) | 70.6 (63.8-78.1) | 71.8 (64.7-79.7) | 71.6 (64.6-79.3) | 0.38 | | 0.63 | | | 1 |  |
| LCER | 70.1 (63.4-77.6) | 71.9 (65.1-79.6) | 67.0 (60.5-74.3) | 73.0 (65.7-81.1) | 0.60 | | 0.71 | | | 1 |  |
| SM | 68.8 (62.2-76.0) | 73.9 (66.8-81.7) | 71.4 (64.5-79.1) | 67.0 (60.4-74.4) | 0.47 | | 0.63 | | | 1 |  |
| CE | 71.2 (64.4-78.8) | 70.8 (64.1-78.2) | 70.7 (63.8-78.4) | 68.6 (61.8-76.2) | 0.47 | | 0.63 | | | 1 |  |
| DAG | 71.5 (64.6-79.1) | 70.7 (63.8-78.3) | 70.7 (63.8-78.4) | 68.6 (61.8-76.2) | 0.45 | | 0.63 | | | 1 |  |
| TAG | 68.2 (61.6-75.6) | 75.6 (68.2-83.7) | 69.3 (62.7-76.7) | 69.2 (62.3-76.9) | 0.65 | | 0.72 | | | 1 |  |
| MAG | 71.7 (64.8-79.3) | 69.4 (62.7-76.7) | 68.3 (61.7-75.7) | 72.5 (65.3-80.4) | 0.48 | | 0.63 | | | 1 |  |
| a. Models were adjusted for age (continuous), age at menarche (continuous), body fat % (continuous), race (non-Hispanic white, non-Hispanic black, other), family history of breast cancer (yes, no), oral contraceptive use (never, less than 1 year, 1-4 years, 5-9 years, more than 10 years), alcohol consumption (never, less than 1 drink per week, 1-2 drinks per week, 3-5 drinks per week, and 6+ drinks per week), parity/age at first birth (nulliparous, 1-2 children & < 25 years, 1-2 children & 25-29 years, 1-2 children & , ≥30 years, ≥3 children & < 25 years, ≥3 children & ≥25 years) and body shape at age 10 (1&2, 3&4, 5, 6-9).  b. Mammographic breast density measures were log_10_ transformed and coefficients were back-transformed (10^β) and bolded if the Bonferroni p-trend is <0.05.  c. Quartile values: PC, Q1=(1165μM-1848μM), Q2=(>1848μM-2097μM), Q3=(>2097μM-2460μM), Q4=(>2460μM-4298μM); LPC, Q1=(60.1μM-144μM), Q2=(>144μM-169μM), Q3=(>169μM-199μM), Q4=(>199μM-312μM); PE, Q1=(76.6-167μM), Q2=(>167-195μM), Q3=(>195-231μM), Q4=(>231-471μM); LPE, Q1=(2.34-4.82μM), Q2=(>4.82-5.84μM), Q3=(>5.84-7.08μM), Q4=(>7.08-12.04μM); PI, Q1=(2.68,-6.44μM), Q2=(>6.44-7.75μM), Q3=(>7.75-9.39μM), Q4=(>9.39-18.77μM); CER, Q1=(3.04-5.58μM), Q2=(>5.58-6.49μM), Q3=(>6.49-7.43μM), Q4=(>7.43-15.57μM); DCER, Q1=(0.879-1.66μM), Q2=(>1.66-1.92μM), Q3=(>1.92-2.25μM), Q4=(>2.25-4.60μM); HCER, Q1=(1.78-3.38μM), Q2=(>3.38-4.02μM), Q3=(>4.02-4.67μM), Q4=(>4.67-10.27μM); LCER, Q1=(1.47-2.92μM), Q2=(>2.92-3.56μM), Q3=(>3.56-4.29μM), Q4=(>4.29-17.27μM); SM, Q1=(354-523μM), Q2=(>523-586μM), Q3=(>586-643μM), Q4=(>643-1118μM); CE, Q1=(1066-1832μM), Q2=(>1832-2062μM), Q3=(>2062-2337μM), Q4=(>2337-4335μM); DAG, Q1=(5.26-16.1μM), Q2=(>16.1-22.1μM), Q3=(>22.1-32.3μM), Q4=(>32.3-106.8μM); TAG, Q1=(276-748μM), Q2=(>748-1018μM), Q3=(>1018-1516μM), Q4=(>1516-6296μM); MAG, Q1=(0.23-0.79μM), Q2=(>0.79-1.71μM), Q3=(>1.71-12.89μM), Q4=(>12.89-56.82μM);  Abbreviations: phosphatidylcholine (PC), lysophosphatidylcholine (LPC), phosphatidylethanolamine (PE), lysophosphatidylethanolamine (LPE), phosphatidylinositol (PI), ceramide (CER), dihydroceramide (DCER), hexosylceramide (HCER), lactosylceramide (LCER), sphingomyelin (SM), cholesteryl ester (CE), diacylglycerol (DAG), triacylglycerol (TAG), monoacylglycerol (MAG), quartiles 1-4 (Q1-4), least square mean (LSM), confidence interval (CI), false discovery rate (FDR), centimeters (cm). | | | | | | | | | | |  |

**Supplementary Figure 2: Covariate-adjusted Proportional Odds Model between the 14 Lipid Sub-pathways and Volumetric Percent Density Categories**


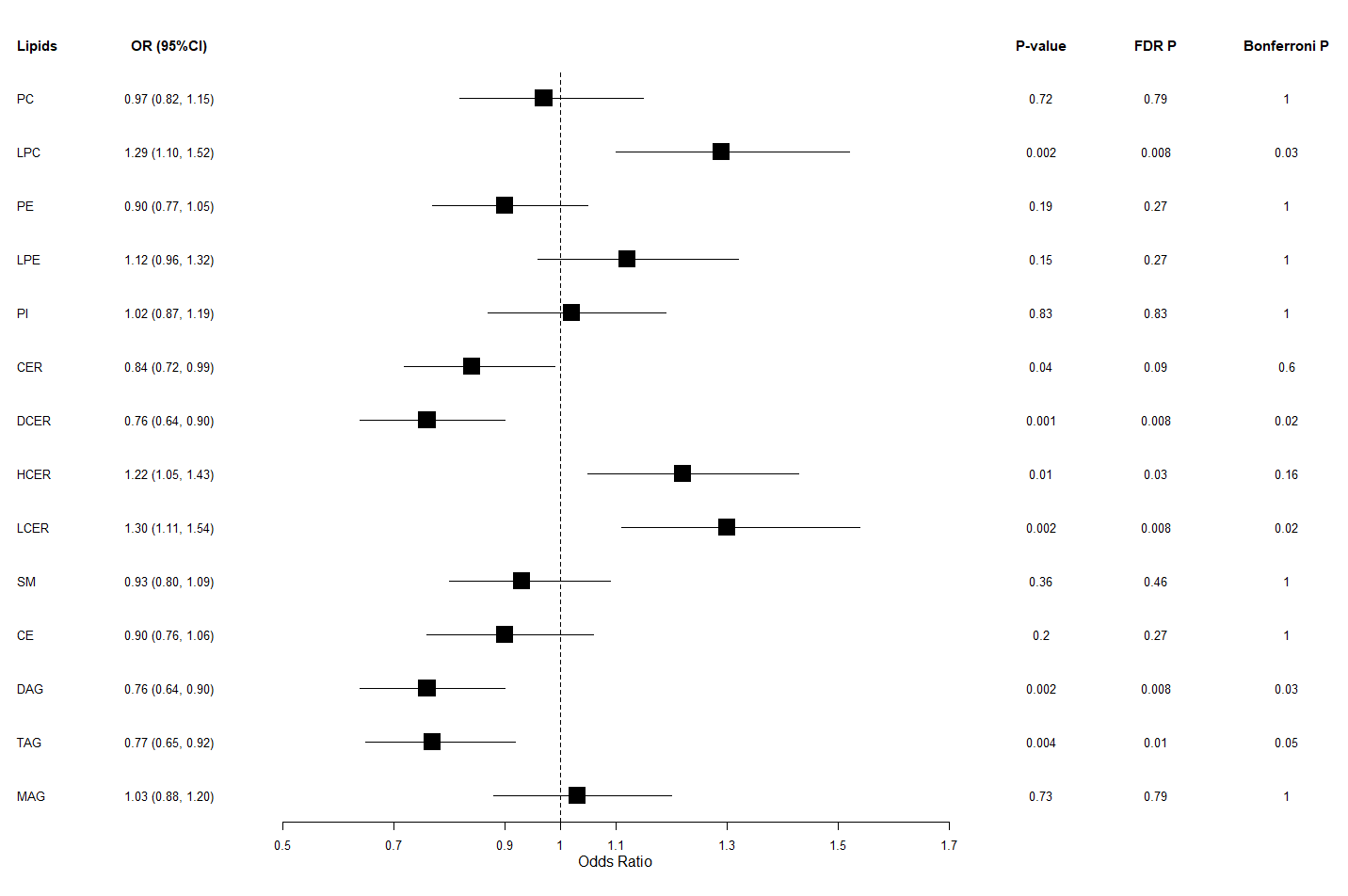


Abbreviations: phosphatidylcholine (PC), lysophosphatidylcholine (LPC), phosphatidylethanolamine (PE), lysophosphatidylethanolamine (LPE), phosphatidylinositol (PI), ceramide (CER), dihydroceramide (DCER), hexosylceramide (HCER), lactosylceramide (LCER), sphingomyelin (SM), cholesteryl ester (CE), diacylglycerol (DAG), triacylglycerol (TAG), monoacylglycerol (MAG), odds ratio (OR), confidence interval (CI), false discovery rate (FDR).
